# Genomic Editing of a Pathogenic Sequence Variant in *ACTA2* Rescues Multisystemic Smooth Muscle Dysfunction Syndrome in Mice

**DOI:** 10.1161/CIRCULATIONAHA.125.074218

**Published:** 2025-05-16

**Authors:** Qianqian Ding, Peiheng Gan, Zhisheng Xu, Hui Li, Lei Guo, Camryn MacDonald, Wei Tan, Efrain Sanchez-Ortiz, John R. McAnally, Yu Zhang, Dileep Karri, Lin Xu, Ning Liu, Eric N. Olson

**Affiliations:** 1 Department of Molecular Biology (Q.D., P.G., Z.X., H.L., C.M., W.T., E.S.-O., J.R.M., Y.Z., D.K., N.L., E.N.O.), University of Texas Southwestern Medical Center, Dallas.; 2 Hamon Center for Regenerative Science and Medicine (Q.D., P.G., Z.X., H.L., C.M., W.T., E.S.-O., J.R.M., Y.Z., D.K., N.L., E.N.O.), University of Texas Southwestern Medical Center, Dallas.; 3 Quantitative Biomedical Research Center, Department of Population and Data Sciences (L.G., L.X.), University of Texas Southwestern Medical Center, Dallas.

**Keywords:** aortic aneurysm, gene editing, muscle, smooth

## Abstract

**BACKGROUND:**

Vascular smooth muscle cells (SMCs), the predominant cell type in the aortic wall, play a crucial role in maintaining aortic integrity, blood pressure, and cardiovascular function. Vascular SMC contractility and function depend on ACTA2 (smooth muscle α-actin 2). The pathogenic variant *ACTA2 c.536G>A* (p.R179H) causes multisystemic smooth muscle dysfunction syndrome, a severe disorder marked by widespread smooth muscle abnormalities, resulting in life-threatening aortic disease and high risk of early death from aneurysms or stroke. No effective treatments exist for multisystemic smooth muscle dysfunction syndrome.

**METHODS:**

To develop a comprehensive therapy for multisystemic smooth muscle dysfunction syndrome, we used CRISPR (clustered regularly interspaced short palindromic repeats)–Cas9 (CRISPR-associated protein 9) adenine base editing to correct the *ACTA2* R179H sequence variant. We generated isogenic human induced pluripotent stem cell lines and humanized mice carrying this pathogenic missense sequence variant. Induced pluripotent stem cell-derived SMCs were evaluated for key functional characteristics, including proliferation, migration, and contractility. The adenine base editor ABE8e-SpCas9-VRQR under control of either an SMC-specific promoter or a cytomegalovirus promoter, and an optimized single guide RNA under control of a U6 promoter were delivered intravenously to humanized R179H mice using adeno-associated virus serotype 9 and phenotypic outcomes were evaluated.

**RESULTS:**

The R179H sequence variant causes a dramatic phenotypic switch in human induced pluripotent stem cell-derived SMCs from a contractile to a synthetic state, a transition associated with aneurysm formation. Base editing prevented this pathogenic phenotypic switch and restored normal SMC function. In humanized mice, the *ACTA2*^R179H/+^ sequence variant caused widespread smooth muscle dysfunction, manifesting as decreased blood pressure, aortic dilation and dissection, bladder enlargement, gut dilation, and hydronephrosis. In vivo base editing rescued these pathological abnormalities, normalizing smooth muscle function.

**CONCLUSIONS:**

This study demonstrates the effectiveness of adenine base editing to treat multisystemic smooth muscle dysfunction syndrome and restore aortic smooth muscle function. By correcting the *ACTA2* R179H sequence variant, the pathogenic phenotypic shift in SMCs was prevented, key aortic smooth muscle functions were restored, and life-threatening aortic dilation and dissection were mitigated in humanized mice. These findings underscore the promise of gene-editing therapies in addressing the underlying genetic causes of smooth muscle disorders and offer a potential transformative treatment for patients facing severe vascular complications.

**Clinical Perspective**

**What Is New?**

- We identified a smooth muscle–specific promoter for vascular gene delivery in vivo.
- We generated a humanized *ACTA2*^R179H/+^ mouse model that recapitulates human multisystemic smooth muscle dysfunction syndrome.
- A single-dose injection of adenine base editor through an adeno-associated virus serotype 9 vector can prevent smooth muscle cell dysfunction and associated pathological manifestations in mice with multisystemic smooth muscle dysfunction syndrome.

**What Are the Clinical Implications?**

- We identified an adenine base-editing strategy with a single guide RNA that is applicable for the human and mouse genomes.
- In vivo gene editing with an adeno-associated virus–CRISPR/Cas9 delivery system represents a potential therapeutic approach for the treatment of human multisystemic smooth muscle dysfunction syndrome and other inherited smooth muscle diseases.

Aortic aneurysm (AA) is characterized by progressive weakening of the aortic wall and permanent arterial dilation.^1^ AA is the second most common disease affecting the aorta after atherosclerosis.^2,3^ AAs are classified into thoracic AA (TAA) and abdominal AA (AAA) based on their anatomic locations. Although AAA and TAA share many features, including vascular smooth muscle cell (vSMC) death, medial elastin breakage or disappearance, extracellular matrix remodeling, and inflammation,^4^ the prevalence of AAA is ≈3 times higher than that of TAA, and TAA has a stronger genetic basis than AAA.^5^ Weakness of the media layers of the aorta eventually results in aortic dissection, which occurs when a tear develops in the inner layer of the aorta, leading to life-threatening intramural hemorrhage.^6,7^ AAs are typically managed with β-adrenergic blocking agents (beta-blockers) and routine surveillance imaging, with surgical repair of the aneurysm when necessary. Acute aortic dissections are often fatal, requiring emergency surgical interventions for survival. Up to 40% of aortic dissection cases result in sudden death.^8^ There is an urgent need to discover novel prevention and treatment strategies for AAs and aortic dissections.

vSMCs, the predominant cell type in the aortic wall, are essential for regulating blood flow, maintaining blood pressure (BP), and preserving aortic integrity. Their dysfunction is a key factor in the development and progression of AAs and aortic dissections. Contraction of vSMCs relies on the cyclic interaction between the thin filaments, composed of the smooth muscle cell (SMC)–specific isoform of α-actin, and the thick filaments, composed of SMC-specific MYH11 (myosin heavy chain 11). Sequence variants in genes encoding vSMC contractile proteins lead to reduced force generation and destabilization of the contractile apparatus, which are key factors contributing to AAs and aortic dissections.^9^ The *ACTA2* gene encodes vascular α-SMA (smooth muscle α-actin), a member of the actin protein family.^10^ The actin monomer encoded by *ACTA2*, called G-actin (globular actin), polymerizes into F-actin (filamentous actin) to allow actin cytoskeleton remodeling.^11^ Missense sequence variants of *ACTA2* account for 14% of inherited ascending thoracic AAs and dissections (TAADs).^12^ Although *ACTA2* is not the predominant actin isoform in visceral organs, variants of *ACTA2* that alter arginine 179 predispose patients to a severe, multisystemic disease known as multisystemic smooth muscle dysfunction syndrome (MSMDS).^13^ Individuals with MSMDS often present with childhood-onset thoracic aortic disease, moyamoya-like cerebrovascular disease, pulmonary hypertension, patent ductus arteriosus, and complications affecting the lungs, liver, gastrointestinal system, bladder, and eyes.^13,14^ The risk of death from aneurysm, stroke, or pulmonary complications is high during childhood. There is no definitive treatment for MSMDS.

CRISPR (clustered regularly interspaced short palindromic repeats)–Cas9 (CRISPR-associated protein 9) genome editing has emerged as a promising approach to correct and potentially cure genetic diseases, particularly those caused by single nucleotide variants.^15–18^ Further development of base editing and prime editing allows for precise correction of nucleotides without introducing DNA double-stranded breaks.^19^ Base editors are fusion proteins consisting of a Cas9 nickase and a deaminase, which are directed by a single guide RNA (sgRNA) to enable targeted base pair conversions within a defined editing window. Two major classes of DNA base editors have been developed: cytosine base editors, which convert a C:G base pair into a T:A base pair, and adenine base editors, which convert an A:T base pair into a G:C base pair.^20,21^ Base editing delivered by adeno-associated viruses (AAVs) has been applied successfully in various cell types and animal models to correct point sequence variants that cause human genetic diseases, directly addressing the root causes of genetic diseases.^16,22–25^

In this study, we used adenine base editing (ABE) to precisely correct the *ACTA2*^R179H^ sequence variant in human induced pluripotent stem cells (iPSCs). In addition, we created a humanized *ACTA2*^R179H^ mouse model that recapitulated human MSMDS phenotypes. Systemic delivery of ABE components through AAV to this mouse model rescued SMC function and prevented MSMDS onset. Our study demonstrates the potential of base editing to treat inherited smooth muscle diseases.

## METHODS

RNA sequencing (RNA-seq) data have been uploaded and deposited at Gene Expression Omnibus (https://www.ncbi.nlm.nih.gov/geo; accession number GSE290426). All data, methods, and materials are available within the article and its Supplemental Material.

### Study Design

The aim of this study was to develop a therapeutic strategy for MSMDS by using CRISPR-Cas9 nucleotide base editing in human cells and a mouse model of MSMDS. Various editing approaches were evaluated, and the most efficient sgRNA was selected to correct the sequence variant. All histological analyses, ultrasound experiments, and BP measurements were conducted and analyzed by blinded operators. Each experiment was performed with biological replicates.

### Study Approval

All experimental procedures involving animals in this study were reviewed and approved by the University of Texas Southwestern Medical Center (UTSW) institutional animal care and use committee. All mice used in this study were housed in the pathogen-free animal resource center at UTSW. All mice were bred inside a specific pathogen-free facility with 12-hour light:dark cycles with a temperature of 18 °C to 24 °C and humidity of 35% to 60% and monitored daily. The mice had no health problems. Use of iPSC lines was reviewed and approved by the UTSW Stem Cell Research Oversight Committee.

### Plasmids and Vector Construction

The pSpCas9(BB)-2A-GFP (PX458) plasmid, a gift from Feng Zhang (Addgene plasmid No. 48138),^26^ was used as the primary scaffold for cloning base editors and Cas9 nickases, as previously described.^16,24,27^ For ABE correction of the *ACTA2*^R179H^ sequence variant in iPSC lines, the sgRNAs (Table S1) were subcloned into engineered vectors using NEBuilder HiFi DNA Assembly (New England Biolabs) to construct all-in-one vectors. For in vivo ABE correction, the N- and C-terminal ABE constructs were modified from Cbh_v5 AAV-ABE N terminus (Addgene plasmid no. 137177) and Cbh_v5 AAV-ABE C terminus (Addgene plasmid No. 137178).^28^

To monitor expression of the base editor within smooth muscle, we generated a series of AAV9 constructs expressing a tdTomato reporter driven by various smooth muscle–specific promoters. These promoters include a 510-bp mouse SM22 promoter fragment (−445 to +65; p445)^29^; a 594-bp chimeric promoter in which a rabbit MyHC enhancer was fused to the SM22 promoter fragment (−440 to +42; p594); a 582-bp chimeric promoter in which the telokin AT/CArG region was fused to the SM22 promoter fragment (−475 to +61; AT-CArG/SM22)^30^; and a 543-bp chimeric promoter in which a fragment of the SM22 promoter (−288 to −116) was fused to the telokin promoter fragment (−190 to +180; SM22/Telokin).^30^ All promoter sequences were synthesized by Integrated DNA Technologies. Cloning was performed using NEBuilder HiFi DNA Assembly into restriction enzyme-digested destination AAV vectors.

### Human iPSC Maintenance and Generation of Isogenic *ACTA2* Variant Cell Lines

Human iPSC lines were generated and maintained as previously described.^31^ In brief, iPSCs were maintained on Matrigel (Corning)–coated 6-well plates in mTeSR plus medium (STEMCELL Technologies) and passaged using Versene (Thermo Fisher Scientific) at ≈70% confluence. One hour before nucleofection, the cells were changed to fresh mTeSR Plus medium supplemented with Rock inhibitor (10 μM). Single-cell suspensions were prepared using Accutase (Innovative Cell Technologies). A total of 8×10^5^ wild-type (WT) iPSCs were nucleofected with a mixture of a single-stranded oligodeoxynucleotide template carrying the target sequence variant and pSpCas9(BB)-2A-GFP (PX458) plasmid containing the sgRNA for exon 6 of *ACTA2*, using the Primary Cell 4D-Nucleofector X kit (Lonza) following the manufacturer’s protocol. Forty-eight hours after nucleofection, GFP^+^ iPSCs were sorted by fluorescence-activated cell sorting and expanded in mTeSR Plus medium supplemented with Rock inhibitor (10 μM) and Primocin (100 μg/mL; InvivoGen). Single GFP^+^ iPSC clones were then picked and genotyped by Sanger sequencing.

### Base Editing in iPSCs

For base editing correction, 8×10^5^ *ACTA2*^R179H/+^ iPSCs were nucleofected with 5 μg of engineered all-in-one vector, as described previously. GFP^+^ iPSCs were sorted by fluorescence-activated cell sorting 48 hours after nucleofection and expanded in mTeSR Plus medium supplemented with Rock inhibitor (10 μM) and Primocin (100 μg/mL; InvivoGen). The cells were cultured for ≈1 week before single clones were picked and genotyped by Sanger sequencing.

### Sanger Sequencing Analysis

Genomic DNA from iPSCs was extracted using Viagen Direct PCR Lysis reagent (Viagen), freshly supplemented with proteinase K (1 μg/μL). PCR amplification of the target sites was performed using PrimeSTAR GXL DNA polymerase (Takara) with the primers listed in Table S2. The PCR products were cleaned up using ExoSap-IT Express (Thermo Fisher Scientific) before undergoing Sanger sequencing on an ABI 3730XL Genetic Analyzer. The sequencing results were analyzed using EditR software to determine editing efficiencies.^32^

### SMC Differentiation

Human iPSCs were differentiated into SMCs as previously described.^33^ In brief, iPSCs were cultured in mTeSR Plus medium on Matrigel-coated plates with daily medium changes until reaching confluence. Differentiation into mesodermal-lineage cells was initiated on day 0 by treating the cells with CHIR99021 (5 μM) and BMP-4 (10 ng/mL) in RPMI1640 medium supplemented with 2% B27. Differentiation into SMCs was initiated on day 3 by culturing the cells with 25 ng/mL VEGF-A and FGFβ in RPMI1640 and 2% B27 minus insulin from day 3 to day 7. Cells were then cultured in RPMI1640 and 2% B27 supplemented with 5 ng/mL PDGF-BB and 2.5 ng/mL transforming growth factor β1 (TGF-β1) from day 7 to day 14, with medium changed every 2 days. The differentiated cells were enriched for SMCs by culturing them in Human Vascular Smooth Muscle Cell Basal Medium and Smooth Muscle Growth Supplement (Gibco).

### Proliferation Assay

For the SMC proliferation assay, a total of 1×10^5^ iPSC–derived SMCs (iPSC-SMCs) were seeded in a Matrigel-coated 12-well plate. When the cells reached ≈70% confluence, they were pulsed with 10 µM 5-EdU for 24 hours. Then, the cells were washed with PBS and fixed with 4% paraformaldehyde in PBS for 10 minutes at room temperature. After fixation, the cells were permeabilized with 0.5% Triton X-100 in PBS for 10 minutes, followed by incubation with the reaction cocktail for 30 minutes at room temperature. After EdU staining, the cells were stained with an antibody against SMA (1:200; Abcam) and counterstained with DAPI (Thermo Fisher Scientific Inc). Imaging was performed using a Keyence BZ-X700 microscope. The analysis was conducted by calculating the average percentage of EdU and α-SMA double-positive cells relative to the total α-SMA–positive cells in the immunofluorescent staining images. For TGF-β1 treatment, SMCs were serum-starved for 24 hours, followed by treatment with TGF-β1 (3 ng/mL) for 48 hours. Subsequent EdU assays were performed.

### Wound-Healing Migration Assay

The wound-healing migration assay was used to assess the migratory ability of SMCs, as previously described.^34,35^ In brief, SMCs were plated in 6-well plates and allowed to grow to confluence followed by 24 hours of starvation. A scratch wound was made across the midline of each well using a sterile 200-µL pipette tip, and suspended cells were washed away with PBS. Human Vascular Smooth Muscle Cell Basal Medium without Smooth Muscle Growth Supplement was added. Images of each well were captured at 0 and 24 hours using a Keyence BZ-X700 microscope. The area covered by cells that migrated across the wound line was quantified using ImageJ software.

### Contraction Assay

The SMC contraction assay was performed using the CytoSelect 24-Well Cell Contraction Assay Kit according to manufacturer instructions (Cell Biolabs). A collagen gel working solution was prepared by mixing collagen solution, 5X medium, and neutralization solution in a cold sterile tube. A total of 5×10^5^ iPSC-SMCs were then mixed with the cold collagen gel working solution and added to the 24-well cell contraction plate. The plate was incubated at 37 ºC and 5% CO_2_ for 1 hour to allow collagen polymerization, followed by adding 1 mL of culture medium with or without contraction inhibitor (2,3-butanedione monoxime). The change in collagen gel size was observed 24 hours later.

### F/G-Actin Isolation

The G-actin/F-actin in vivo assay kit (Cytoskeleton) was used to isolate both G-actin and F-actin fractions from SMCs. In brief, SMCs were lysed using Lysis and F-actin Stabilization Buffer for 10 minutes at 37 °C, followed by centrifugation at 350×*g* for 5 minutes to pellet unbroken cells and debris. The supernatant was transferred into ultracentrifuge tubes and centrifuged at 100 000×*g* at 37 °C for 1 hour. Soluble actin (G-actin) was collected from the supernatant. The insoluble F-actin in the pellet was resuspended in F-actin depolymerization buffer and incubated on ice for 1 hour, with gentle mixing every 15 minutes to convert F-actin into soluble G-actin. Samples from the supernatant (G-actin) and pellet (F-actin) fractions were then analyzed by Western blotting using an actin antibody provided in the kit.

### Western Blot Analysis

Proteins were isolated using RIPA buffer (Sigma-Aldrich) supplemented with protease and phosphatase inhibitors (Roche). Samples were then centrifuged for 15 minutes at 10 000×*g* at 4 ºC and supernatant was stored at −80ºC. Protein concentration was measured with a bicinchoninic acid assay (Thermo Fisher Scientific) and equal amounts of protein were loaded on a Mini-PROTEAN TGX gel (Bio-Rad). Proteins were transferred on a polyvinylidene fluoride membrane (Millipore), blocked in 5% milk in TBS-Tween 0.1%, and incubated at 4 ºC overnight with the primary antibody: anti–α-SMA (Abcam; 1:1000), anti-Transgelin (SM22; Abcam; 1:1000), or anti-vinculin (Sigma-Aldrich; 1:20000). Secondary antibodies were horseradish peroxidase–conjugated goat anti-rabbit (Bio-Rad; 1:3000) and horseradish peroxidase–conjugated goat anti-mouse (Bio-Rad; 1:3000), which were incubated for 1 hour at room temperature. Immunodetection was done on a ChemiDoc MP Imaging System (Bio-Rad) using Western Blotting Luminol Reagent (Santa Cruz Biotechnology). Mean densitometric analysis was performed using ImageJ.

### Quantitative Real-Time PCR Analysis

Total RNA was extracted, and cDNA was reverse transcribed using iScript reverse transcription supermix (Bio-Rad Laboratories) according to manufacturer protocol. For quantitative reverse transcription polymerase chain reaction, gene expression was measured using KAPA SYBR FAST Master mix. GAPDH was used as the housekeeping gene. Quantitative reverse transcription polymerase chain reaction primers are listed in Table S2.

### Off-Target Analysis

Deep amplicon sequencing was used to assess potential off-target editing by ABE8e-VRQR and sgRNA3 in iPSCs. The top 8 candidate off-target sites in the human genome were identified using the cutting frequency determination score from CRISPOR.^36,37^ Genomic DNA from iPSCs was extracted as described previously. A first round of PCR was performed to add a 67-bp adaptor sequence (forward: TCGTCGGCAGCGTCAGATGTGTATAAGAGACAG; reverse: GTCTCGTGGGCTCGGAGATGTGTATAAGAGACAG) to the target PCR amplicon. A second round of PCR was conducted to add Illumina flow cell binding sequences and barcodes. Primers are listed in Table S2.

### Generation of *ACTA2*^R179H^ Knock-In Mice

*ACTA2*^R179H^ knock-in mice were generated by microinjection of zygotes with a mixture of Cas9 mRNA, sgRNA, and single-stranded oligodeoxynucleotide, following a modified protocol.^38^ Female B6C3F1 mice (6 weeks old) were treated for superovulation and mated with male B6C3F1 mice to induce zygote production. F0 mosaic mice were genotyped, and positive founders were bred with C57BL/6N mice for germline transmission. F1 knock-in mice were confirmed by Sanger sequencing. Both male and female mice were included in this study.

### Systemic AAV9 Delivery in vivo

WT C57BL/6N mice at postnatal day 3 (P3) were injected through the facial vein with 20 µL AAV9 (9×10^13^ vg/kg) expressing a tdTomato reporter driven by different smooth muscle–specific promoters using an ultrafine BD insulin syringe (Becton Dickinson). *ACTA2*^R179H/+^ mice at P3 were injected through the facial vein with 20 µL of AAV9-containing N-terminal and C-terminal ABE8e-VRQR-SpCas9-sgRNA (total of 9×10^13^ vg/kg) using an ultrafine BD insulin syringe (Becton Dickinson).

### Ultrasound Imaging of the Thoracic Aorta

Transthoracic 2-dimensional ultrasound was conducted to assess aortic morphology using a VisualSonics Vevo2100 imaging system.^39^ During the imaging, mice were anesthetized with 1% to 3% isoflurane (vol/vol) and placed on a heating platform to minimize procedural stress and prevent hypothermia. The maximum internal diameter of the ascending thoracic aorta was measured using longitudinal images of the aortic arch. All measurements were performed by an operator blinded to the study.

### BP Measurements

BP of mice was measured using a noninvasive small animal BP monitoring system (CODA Monitor).^40^ In brief, the system parameters and corresponding experimental series were set on the detection software interface. Mice were placed in a holder in a quiet room and warmed on a heating platform. An occlusion tail cuff was gently placed through the tail root, and a VPR sensor cuff was positioned within 2 mm of the occlusion cuff. The mice were allowed ≥5 minutes to acclimate to their surroundings. Three days of acclimation measurements followed by another 2 days of data collection measurements were conducted to ensure accurate readings. All measurements were performed by an operator blinded to the study.

### Bulk RNA-Seq Analysis

Total RNA from iPSC-SMCs was extracted by using TRIzol (Thermo Fisher Scientific). RNeasy Mini Kit (Qiagen) was used to purify RNA following manufacturer recommendations. RNA-seq libraries were prepared using the KAPA mRNA HyperPrep kit (Kapa Biosystems) according to manufacturer instructions. High-output, 50-cycle pair-ended sequencing was performed using an Illumina NextSeq2000 sequencer at the UTSW CRI Sequencing Facility. For the bulk RNA-seq analysis, adaptors were trimmed using Trim Galore (v0.6.4). The trimmed reads were aligned to the human genome (GRCh38) using STAR (v2.7.3a). Gene expression quantification was performed using the featureCounts function from the Subread package (v1.6.3). Genes with 0 expression in >30% of samples were filtered out before downstream analysis. Expression data were normalized using the Voom method from the limma package (v3.50.3), which was also used to identify differentially expressed genes.

### Angiotensin II Infusion

Male wild-type (WT), saline-treated *ACTA2*^R179H/+^, and AAV9-SM22/Telokin-ABE–treated *ACTA2*^R179H/+^ mice (4 mice for each group) were subcutaneously administered angiotensin II (Ang II; 1 μg·kg·min) for 2 weeks by using the implanted osmotic pump (Alzet model 2002).^41^ After the Ang II infusion, aortic diameters were measured by ultrasound. Aortas were harvested and histological analyses were performed.

### Histological Analysis

Aortas were harvested and fixed in 4% paraformaldehyde in PBS overnight, followed by dehydration in 30% sucrose, and embedded in a 1:1 solution of 30% sucrose:tissue freezing medium. Blocks of embedded tissues were cryosectioned to a thickness of 10 µm and stored at −80 °C. For immunostaining, selected slides were rewarmed at room temperature and the tissues were outlined with a hydrophobic PapPen barrier. Next, the slides were washed in PBS twice and blocked for 1 hour at room temperature in a solution of 10% normal goat serum, 0.3% Triton X-100, and 1% BSA in PBS. After blocking, primary antibodies were diluted in blocking solution and incubated on the tissue overnight at 4 °C. The primary antibody used in this experiment was α-SMA (Abcam; 1:200). The following day, the slides were washed at room temperature with PBST and incubated for 1 hour at room temperature with secondary antibody (goat anti-mouse AlexaFluor488; Invitrogen; 1:200) and mounted using Prolong Gold Antifade Reagent (Cell Signaling Technology). Images were captured on a Zeiss LSM 800 Confocal Laser Scanning microscope. The aorta sections were also subjected to Hart staining or Masson's trichrome for elastin and collagen staining. Small intestine, bladder, and kidney were dissected and fixed in 4% paraformaldehyde in PBS overnight, followed by dehydration in 70% ethanol and paraffin embedding. Serial sections were cut and mounted on slides, followed by hematoxylin & eosin staining. Images were captured on a Keyence BZ-X700 microscope.

### Mouse DNA Sequencing Analysis

Deep amplicon sequencing with MiSeq V3 600 cycles was used to measure the on-target editing efficiency for *ACTA2* in tissues from mice treated with and without ABE. Genomic DNA from mice was extracted using DNeasy Blood & Tissue Kit (Qiagen). PCR products from genomic DNA were generated using PrimeSTAR GXL DNA polymerase (Takara) with the primers listed in Table S2.

### Statistical Analysis

All data are presented as mean±SEM. The Shapiro-Wilk normality test was used to assess normality. If a variable was not normally distributed, or if the sample size was too small to assess normality, nonparametric tests were applied. For comparisons between 2 groups, an unpaired, 2-tailed Student *t* test was used for normally distributed data; the Mann-Whitney test was used for non-normally distributed data. For comparisons involving >2 groups, 1-way or 2-way ANOVA followed by Tukey multiple comparisons test was used for normally distributed data. For non-normally distributed data, the Kruskal-Wallis test followed by Dunn multiple comparisons test was applied. Data analyses were performed with GraphPad Prism software (version 10.4.2). *P* values <0.05 were considered statistically significant.

## RESULTS

### ABE of the *ACTA2*^R179H^ Variant in Human iPSCs

The *ACTA2*^R179H^ sequence variant (c.536G>A) is caused by a nucleotide substitution from guanine to adenine, resulting in an amino acid change from arginine to histidine, which can be edited precisely using ABE. To identify optimal CRISPR-Cas9 base editing components for correcting the *ACTA2*^R179H^ sequence variant, we first deployed homology-directed repair to generate human iPSC lines carrying the pathogenic missense sequence variant from healthy control iPSCs. We established an isogenic heterozygous clone (*ACTA2*^R179H/+^) that recapitulates the heterozygous genotype identified in patients and an isogenic homozygous clone (*ACTA2*^R179H/R179H^) that has not been previously reported in patients (Figure S1).

**Figure 1.**
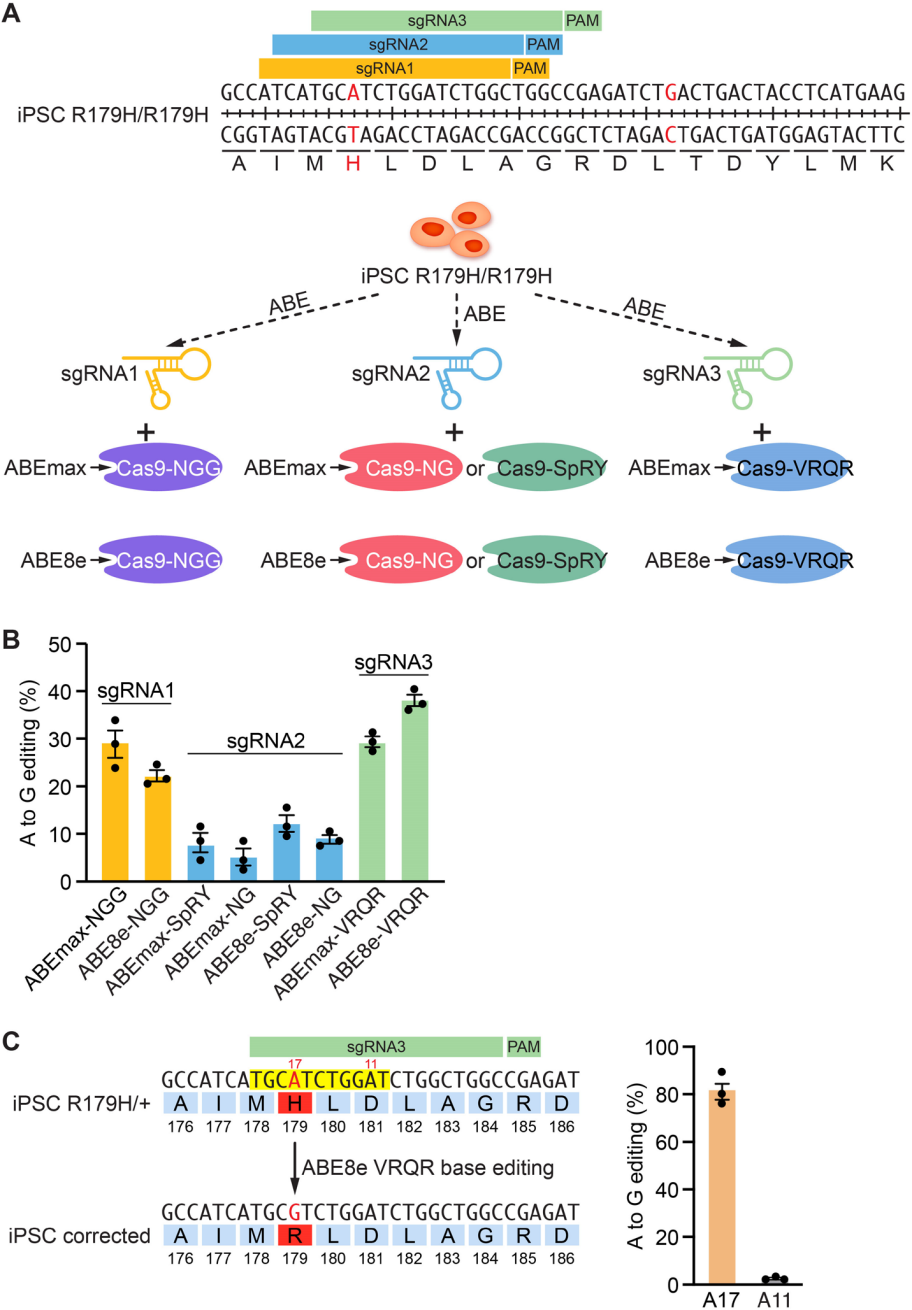
**In vitro optimization of the adenine base editing system to correct the pathogenic *ACTA2* R179H sequence variant. A**, Schematic diagram of 3 sgRNAs and 8 candidate base editor variants that were evaluated for their efficiencies in correcting the pathogenic adenine (A) to guanine (G) within the human R179H/R179H iPSC line. **B**, DNA editing efficiency at the target site in R179H/R179H iPSCs was assessed by Sanger sequencing 72 hours after transfection. **C**, DNA editing efficiency in sorted R179H/+ iPSCs was assessed by Sanger sequencing. Sequences of sgRNA3 and PAM are shown. The editing window for ABE8e-SpCas9-VRQR is highlighted in yellow. The positions of the target A (A17) and the bystander A (A11) are indicated, with nucleotide counting beginning immediately 5′ of the PAM sequence. The bar graph shows the A-to-G editing efficiency in the sorted R179H/+ iPSC line. The on-target site (A17) is indicated in orange; the bystander site (A11) is shown in gray. Data are presented as mean±SEM of 3 replicates. ABE indicates adenine base editing; iPSC, induced pluripotent stem cell; PAM, protospacer adjacent motif; and sgRNA, single guide RNA .

To identify an optimal base editing strategy, we fused 2 engineered deaminases–ABEmax and ABE8e–with different variants of SpCas9 nickase: SpCas9-NG, which targets an NG protospacer adjacent motif; SpRY, which targets an NRN protospacer adjacent motif; and SpCas9-VRQR, which targets an NGA protospacer adjacent motif. We screened these ABEs for their efficiency of correction of our *ACTA2*^R179H/R179H^ iPSC line through transient transfection with each of the 3 sgRNAs, respectively (Figure 1A). The results showed that sgRNA3 combined with ABE8e-SpCas9-VRQR had the highest editing efficiency at the on-target site (Figure 1B). We then introduced the combination of sgRNA3 and ABE8e-SpCas9-VRQR into the *ACTA2*^R179H/+^ iPSC line through plasmid nucleofection, followed by fluorescence-activated cell sorting of GFP^+^ cells, and obtained high efficiency of A-to-G editing at the on-target site (81.67%) with little bystander editing (2.50%) (Figure 1C; Figure S2). To evaluate possible off-target editing, we further performed genomic deep sequencing analysis of the sorted GFP^+^ cells. We observed minimal to no off-target DNA editing (0.1% or less) at all adenine bases for 8 tested candidate off-target loci, which were identified using the bioinformatic tool CRISPOR^37^ (Figure S3). After genomic editing, the corrected iPSC line displayed a normal karyotype (Figure S4). These results demonstrated that sgRNA3 with ABE8e-SpCas9-VRQR can efficiently and precisely correct the target pathogenic missense variant with minimal bystander editing and little to no DNA editing at tested off-target sites.

### Functional Analyses of ABE-Edited Human iPSC-SMCs

Sequence variants in genes encoding smooth muscle contractile proteins impair the normal functions of vSMCs, leading to a switch from a quiescent, contractile phenotype to a proliferative, synthetic, and migratory phenotype.^42,43^ This phenotypic switch of vSMCs has been observed in AAA, TAA, and TAAD in both human and rodent studies. vSMCs derived from patients with heterozygous *ACTA2* sequence variants exhibit significantly increased proliferation compared with control cells.^44^ To determine whether the *ACTA2*^R179H^ sequence variant causes phenotypic switching of SMCs, we differentiated WT, *ACTA2*^R179H/+^, and *ACTA2*^R179H/R179H^, as well as the corrected human iPSC lines (all derived from single clones), into SMCs and evaluated their proliferation, migration, and contractility.^33^ iPSCs from all 4 groups differentiated readily into SMCs, as confirmed by the expression of multiple SMC-specific genes (Figure S5).

**Figure 2.**
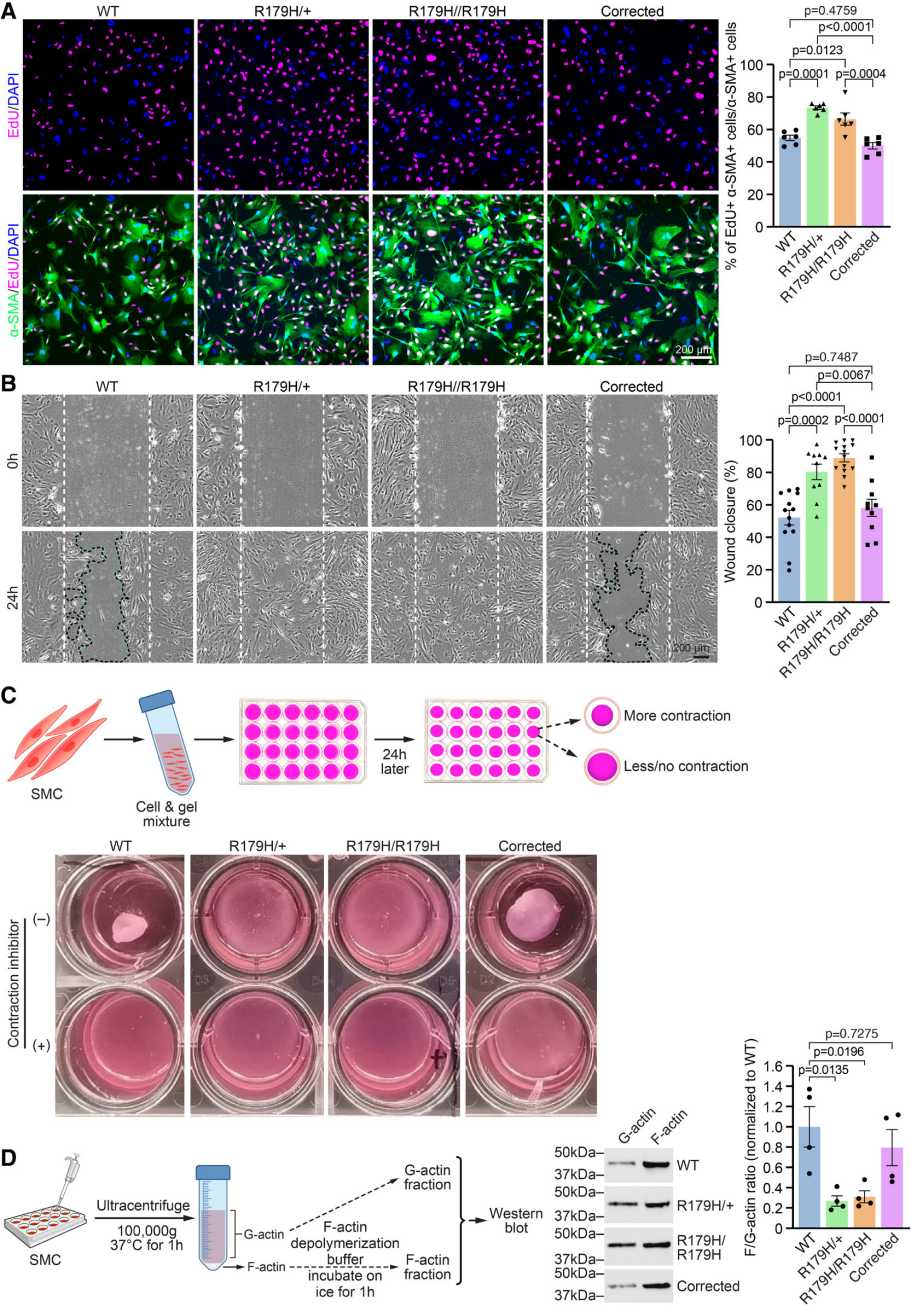
**Adenine base editing corrects the pathogenic phenotypes in human iPSC-SMCs with the *ACTA2* R179H sequence variant. A**, EdU assays were performed on WT, R179H/+, R179H/R179H, and ABE–corrected R179H/+ iPSC-SMCs. Cells were stained for smooth muscle α-actin (α-SMA; green), EdU (purple), and DAPI (blue). The percentage of EdU^+^ and α-SMA^+^ cells, relative to the total number of α-SMA^+^ cells, was quantified and displayed on the right (n=6). **B**, Serum-starved WT, R179H/+, R179H/R179H, and ABE-corrected R179H/+ iPSC-SMCs were subjected to a scratch and the migration responses were assessed 24 hours later. The area covered by cells that migrated across the wound line was quantified and displayed on the right (WT [n=13], R179H/+ [n=10], R179H/R179H [n=14], ABE-corrected [n=10]). **C**, Collagen gel contraction assay shows the contractility of WT, R179H/+, R179H/R179H, and ABE-corrected R179H/+ iPSC-SMCs. In the presence of the contraction inhibitor 2,3-butanedione monoxime, the contraction of all groups was prevented (n=3). **D**, Ultracentrifugation-based F/G-actin isolation followed by Western blot analysis showing the F- and G-actin fractions. The F/G-actin ratio was quantified and displayed on the right (n=4). Data are presented as mean±SEM. Statistical comparisons are based on 1-way ANOVA post hoc corrected by Tukey. Representative images reflect the most typical or consistent results observed across replicates. ABE indicates adenine base editing; F-actin, filamentous actin; G-actin, globular actin; iPSC-SMCs, induced pluripotent stem cell-derived smooth muscle cells; and WT, wild-type.

We first conducted EdU incorporation assays to assess SMC proliferation.^45^ The SMCs were pulsed with 5-EdU for 24 hours and subsequently costained with an antibody against α-SMA. We then calculated the percentage of EdU and α-SMA double-positive cells relative to total α-SMA–positive cells. The results indicated a 30% increase in EdU incorporation in *ACTA2*^R179H/+^ iPSC-SMCs compared with WT iPSC-SMCs, and a 20% increase in *ACTA2*^R179H/R179H^ iPSC-SMCs compared with WT iPSC-SMCs. Conversely, corrected iPSC-SMCs showed a 40% and 30% reduction in EdU incorporation compared with *ACTA2*^R179H/+^ iPSC-SMCs and *ACTA2*^R179H/R179H^ iPSC-SMCs, respectively (Figure 2A). There was no significant difference between corrected and WT iPSC-SMCs, demonstrating that ABE correction reduced the abnormally high proliferation of variant iPSC-SMCs (Figure 2A).

SMCs are highly sensitive to several growth factors, which regulate SMC phenotypes. TGF-β promotes SMC differentiation, converting synthetic SMCs into contractile SMCs.^46^ To further investigate whether the R179H sequence variant causes different responses to stimuli, we treated SMCs with TGF-β1 for 48 hours, followed by an EdU assay. Exposure to TGF-β1 reduced the proliferation of SMCs across all 4 groups compared with unstimulated cells (Figure S6), indicating a phenotype shift toward contractile SMCs. No difference in proliferation was observed among all 4 TGF-β1–treated groups. One possible explanation is that these SMCs are derived from iPSCs and may not have fully differentiated into mature SMCs, thereby failing to completely recapitulate their functional capacities. Notably, in a recent study of another ACTA2 sequence variant, R179C, TGF-β1 treatment increased the expression of contractile proteins to similar levels in both WT and variant groups.^47^

We next performed wound-healing migration assays to evaluate the migratory capacity of SMCs. SMCs were seeded in 6-well plates and allowed to grow to full confluence. A scratch wound was made across the midline of each well, and images were captured at 0 and 24 hours after wounding. The area covered by cells migrating across the wound line was then quantified. As shown in Figure 2B, *ACTA2*^R179H/+^ iPSC-SMCs exhibited a 50% increase in migration compared with WT iPSC-SMCs, and *ACTA2*^R179H/R179H^ iPSC-SMCs displayed a 70% increase in migration relative to WT iPSC-SMCs. In contrast, corrected iPSC-SMCs showed a 40% and 50% reduction in the covered area compared with *ACTA2*^R179H/+^ iPSC-SMCs and *ACTA2*^R179H/R179H^ iPSC-SMCs, respectively. No significant difference was observed between corrected and WT iPSC-SMCs (Figure 2B).

Contractility of vSMCs within the aortic wall is vital for maintaining vSMC/extracellular matrix integrity and plays a key role in mechanotransduction. Impaired vSMC contractility has been observed in patients with AAA compared with healthy vSMCs.^1^ We evaluated cell contractility by mixing a freshly polymerized collagen matrix with SMCs. Changes in collagen gel size were observed 24 hours later. WT iPSC-SMCs exhibited robust contraction, as evidenced by a reduced gel area; both *ACTA2*^R179H/+^ and *ACTA2*^R179H/R179H^ iPSC-SMCs displayed minimal contraction (Figure 2C). The corrected iPSC-SMCs displayed a gel area comparable with that of WT iPSC-SMCs. In the presence of the contraction inhibitor 2,3-butanedione monoxime, the contraction of all groups was prevented (Figure 2C).

Smooth muscle contraction depends on actin polymerization. In vitro assays have demonstrated that the R179H actin sequence variant disrupts actin polymerization, and cells expressing R179H actin have increased levels of monomeric G-actin.^48^ To assess actin polymerization, we isolated both F-actin and G-actin from iPSC-SMCs and performed Western blot analysis.^49^ *ACTA2*^R179H/+^ and *ACTA2*^R179H/R179H^ iPSC-SMCs showed a markedly decreased F-actin to G-actin ratio compared with WT SMCs, whereas ABE correction restored the normal F-actin to G-actin ratio (Figure 2D). Taken together, these findings suggest that the R179H sequence variant drives a phenotypic switch in SMCs from a contractile to a synthetic state, a process associated with aneurysm formation, and ABE correction effectively normalizes these phenotypic alterations.

### Normalization of Gene Expression in ABE-Corrected iPSC-SMCs

**Figure 3.**
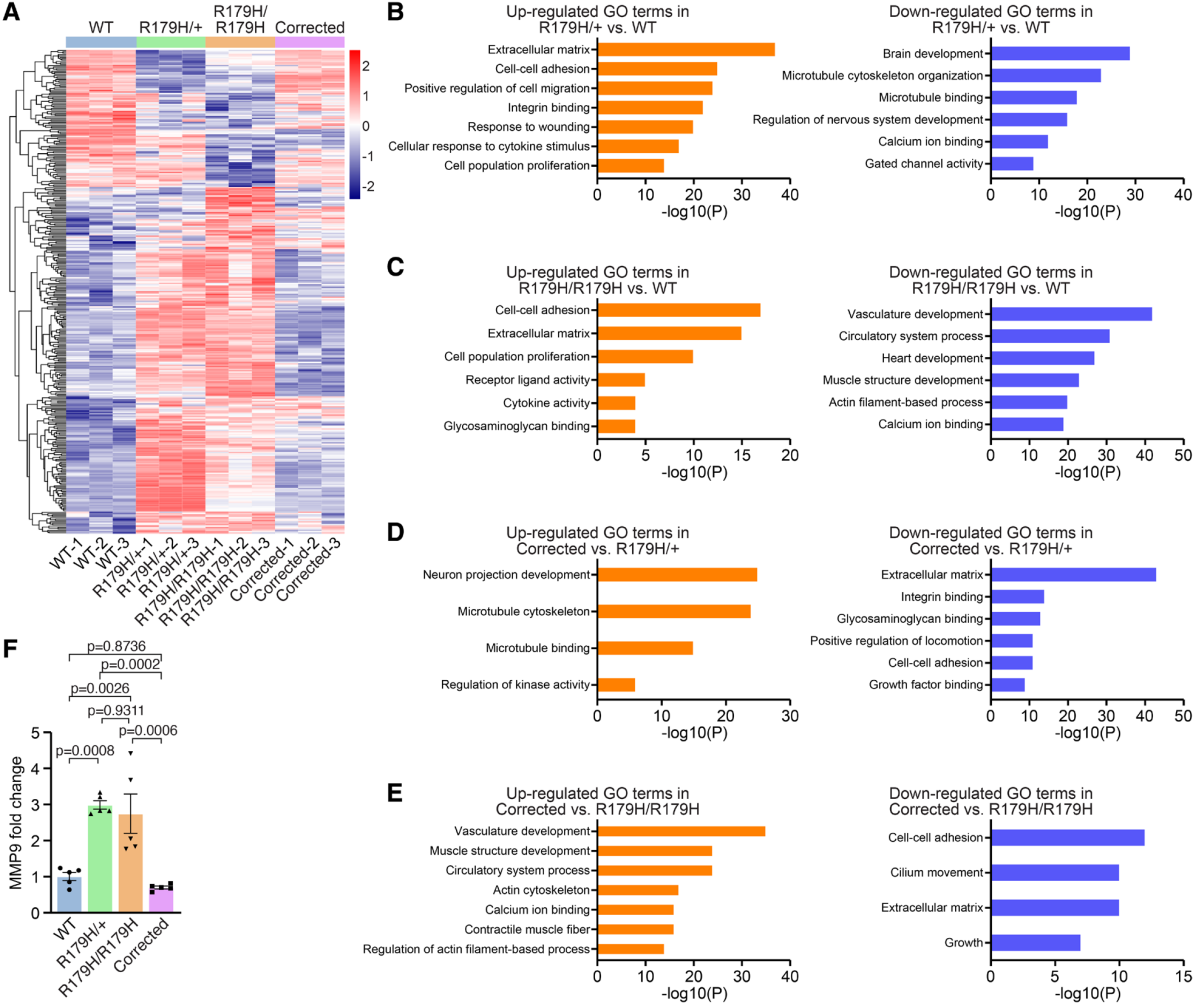
**Adenine base editing normalizes transcriptional profiles of *ACTA2* R179H sequence variant iPSC-SMCs. A**, Heatmaps of the differentially expressed genes in WT, R179H/+, R179H/R179H, and ABE–corrected R179H/+ iPSC-SMCs (n=3). GO terms associated with the differentially expressed genes in the comparison of R179H/+ versus WT iPSC-SMCs (**B**), R179H/R179H versus WT iPSC-SMCs (**C**), ABE-corrected R179H/+ versus R179H/+ iPSC-SMCs (**D**), and ABE-corrected R179H/+ versus R179H/R179H iPSC-SMCs (**E**) are shown. **F**, Relative expression of MMP-9 in WT, R179H/+, R179H/R179H, and ABE-corrected R179H/+ iPSC-SMCs (n=5). The quantitative data are presented as mean±SEM. Statistical comparisons are based on 1-way ANOVA post hoc corrected by Tukey. ABE indicates adenine base editing; GO, Gene Ontology; iPSC-SMC, induced pluripotent stem cell-derived smooth muscle cell; MMP-9, matrix metalloproteinase-9; and WT, wild-type .

We next assessed transcriptome-wide changes in variant and ABE-corrected SMCs using RNA-seq. Differentially expressed genes were analyzed and heatmaps showed that the transcriptome profiles of ABE-corrected SMCs more closely resembled those of WT SMCs than the profiles of *ACTA2*^R179H/+^ and *ACTA2*^R179H/R179H^ SMCs (Figure 3A). Gene Ontology analyses showed increased regulation of the extracellular matrix, cell–cell adhesion, and cell proliferation, alongside decreased regulation of cytoskeleton organization in *ACTA2*^R179H/+^ SMCs compared with WT SMCs (Figure 3B). Gene Ontology analysis comparing *ACTA2*^R179H/R179H^ and WT SMCs revealed dysregulation in extracellular matrix organization and vasculature development (Figure 3C). These abnormalities were partially reversed after ABE correction (Figure 3D and 3E). In addition, we observed that expression of matrix metalloproteinase-9, a key enzyme in vSMC phenotype switching and AA formation, was 2.9-fold higher in *ACTA2*^R179H/+^ and 2.7-fold higher in *ACTA2*^R179H/R179H^ SMCs compared with WT SMCs (Figure 3F). In contrast, matrix metalloproteinase-9 expression in ABE-corrected SMCs was not significantly different from that of WT SMCs (Figure 3F).

To dissect the heterogeneity in gene expression and cell phenotypes at the single-cell level, we compared our bulk RNA-seq data with published single-cell RNA-seq data sets.^46,50^ We observed increased expression of markers of the synthetic SMC population, such as *MMP-9*, *VIM*, *COL1A1*, and *GJA1*, in *ACTA2*^R179H/+^ iPSC-SMCs compared with WT iPSC-SMCs (Figure S7). The corrected iPSC-SMCs exhibited expression of these synthetic markers similar to those of WT iPSC-SMCs, indicating normalization of the synthetic SMC phenotype. Fibromyocytes are a modulated type of SMC commonly found in AAs.^51,52^ They exhibit properties of both fibroblasts and muscle cells, expressing higher levels of collagen and proteoglycan-related genes compared with other SMC clusters. We found that *ACTA2*^R179H/+^ iPSC-SMCs exhibited marked upregulation of fibromyocyte marker genes, including *SPP1*, *FN1*, *LUM*, *TIMP1*, and *TNFRSF11B*. Corrected iPSC-SMCs demonstrated expression patterns of these markers that closely resembled those of WT iPSC-SMCs, indicating a reversion toward the normal SMC phenotype (Figure S7). These data suggest that our ABE system effectively normalizes gene expression and cell states in variant SMCs.

### Development of a Humanized Mouse Model of MSMDS

**Figure 4.**
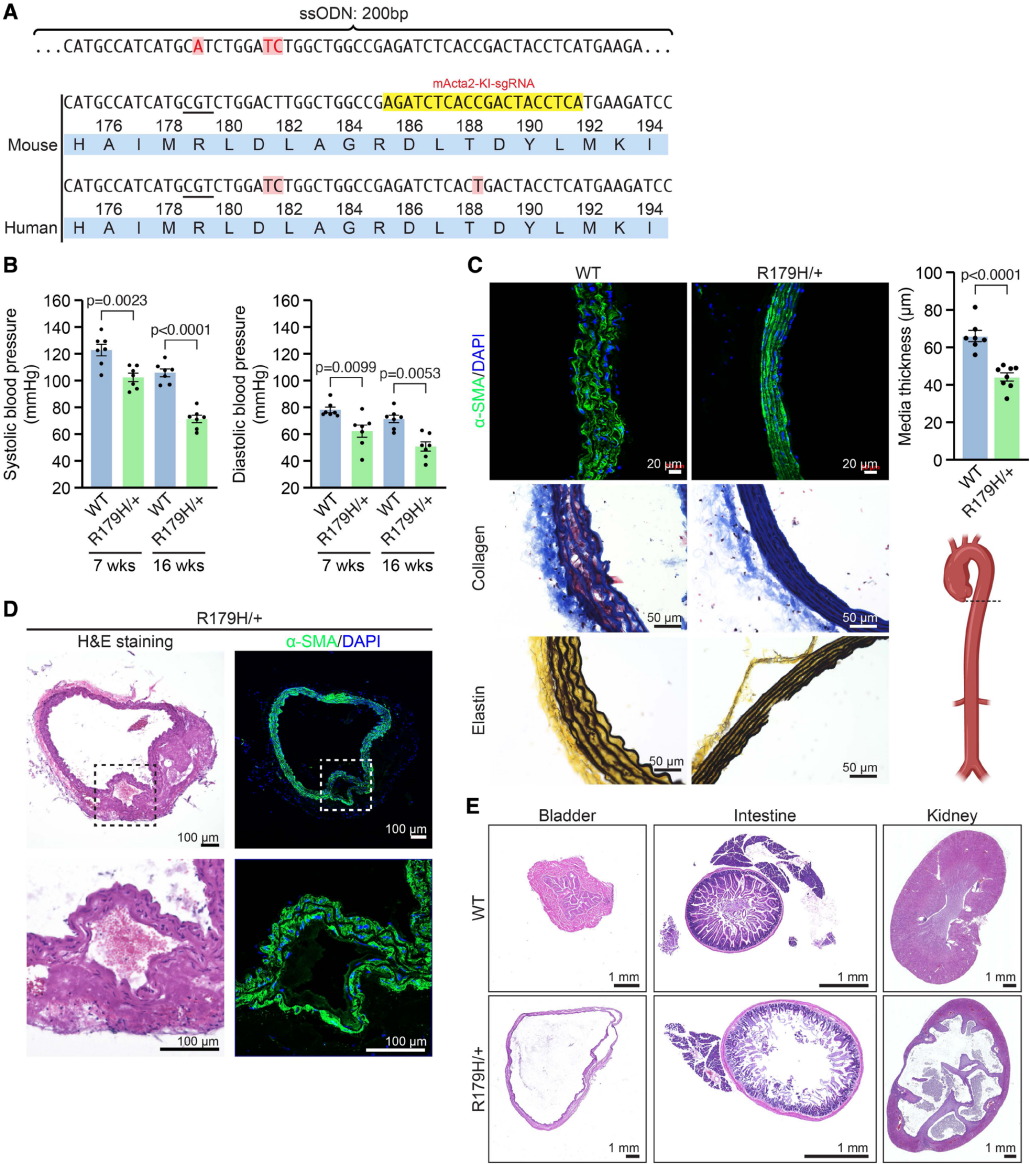
**Generation of a humanized MSMDS mouse model. A**, A humanized MSMDS mouse model was generated by replacing part of the murine *Acta2* genomic sequence with the human *ACTA2* sequence containing the p.R179H variant. This was achieved through CRISPR–Cas9 homology-directed repair, using a knock-in sgRNA (highlighted in yellow), and an ssODN donor template (the pathogenic sequence variant and 2 human nucleotides are highlighted in red). A comparison of mouse and human *ACTA2* nucleotide and amino acid sequences is also provided. Codon for R179 (CGT) is underlined. **B**, Systolic and diastolic blood pressures of *ACTA2*^R179H/+^ and WT mice at 7 weeks and 16 weeks of age, respectively (n=7). **C**, Top, Representative images of transverse sections of thoracic aorta stained with α-SMA (green) and DAPI (blue), along with quantification of the media thickness. Middle: representative Masson's trichrome staining demonstrating collagen deposition (blue). Bottom: representative images of Hart staining demonstrating elastin integrity (black). The schematic graph of the aorta indicates the location where sections were taken for the histological analysis of the aorta. WT and *ACTA2*^R179H/+^ mice are analyzed at ≈24 weeks of age (WT [n=7], R179H/+ [n=8]). **D**, Representative images of hematoxylin & eosin staining and immunofluorescence staining of *ACTA2*^R179H/+^ mice at ≈24 weeks of age, showing aortic dissection. The areas highlighted by the squares are shown in an enlarged view below. **E**, Representative H&E staining of bladder (left), small intestine (middle), and kidney (right) of WT and *ACTA2*^R179H/+^ mice at ≈24 weeks of age. The quantitative data are presented as mean±SEM. The Mann-Whitney test was used to compare diastolic blood pressure at 7 weeks of age; Student *t* test was used for all other comparisons. Representative images reflect the most typical or consistent results observed across replicates. α-SMA indicates smooth muscle α-actin; Cas9, CRISPR-associated protein 9; CRISPR, clustered regularly interspaced short palindromic repeats; KI, knock-in; MSMDS, multisystemic smooth muscle dysfunction syndrome; sgRNA, single guide RNA; ssODN, single-stranded oligodeoxynucleotide; and WT, wild-type.

Encouraged by the findings in human iPSC-SMCs, we further investigated whether the base editing strategy developed for the human genome could be applied to a mouse model of human MSMDS. Although the amino acids are identical between human ACTA2 and mouse Acta2, the DNA sequence encoding the R179 region of the protein is different. Thus, sgRNAs and editing strategies developed for the human genome are not directly applicable to mice. To perform preclinical studies using our human sequence–specific base editing strategy, we generated partially humanized mice containing the *ACTA2* c.536G>A (p.R179H) human missense variant within exon 6 of the mouse *Acta2* gene, allowing for the testing of human genome–specific ABE strategies. In brief, a 200-bp nucleotide oligo containing the human *c.536G>A* missense sequence variant along with 2 downstream human nucleotides was co-injected with a knock-in sgRNA into mouse zygotes to generate the partially humanized R179H mouse line (Figure 4A).

The humanized mice were viable, but ≈40% of these mice (both male and female) were infertile. When crossing 2 heterozygous parents, we obtained both heterozygous and homozygous offspring; however, the proportion of homozygous offspring (≈10%) was lower than the expected mendelian ratio of 25%. Given that patients with the R179H sequence variant are heterozygous, we opted to use *ACTA2*^R179H/+^ mice for our subsequent studies. These *ACTA2*^R179H/+^ mice recapitulated many of the phenotypes observed in patients with the same sequence variant. The *ACTA2*^R179H/+^ mice displayed significantly reduced systolic and diastolic BPs as early as 7 weeks of age compared with their littermate controls (Figure 4B). Thoracic aortas from the *ACTA2*^R179H/+^ mice were harvested at ≈24 weeks of age and tissue sections were subjected to histological analysis to characterize the aortic lesions using immunofluorescence, Hart staining (for elastin), and Masson's trichrome staining (for collagen). Immunofluorescence staining with α-SMA revealed a reduction in media thickness in *ACTA2*^R179H/+^ mice compared with WT mice, indicating weakness of the media layers of the aorta in *ACTA2*^R179H/+^ mice (Figure 4C). Masson's trichrome staining revealed increased collagen deposition (blue) within the medial layer of *ACTA2*^R179H/+^ aortas, along with noticeable elastin fiber (black) fragmentation in the aortas of *ACTA2*^R179H/+^ mice (Figure 4C). Notably, ≈30% (5 out of 16) of *ACTA2*^R179H/+^ mice exhibited tears in the middle aortic layer by 24 weeks of age, as evidenced by hematoxylin & eosin and immunofluorescence staining with α-SMA, although no visible aneurysms or bleeding were observed during aorta harvest in some of these mice (Figure 4D).

*ACTA2*^R179H/+^ mice also exhibited systemic abnormalities indicative of visceral smooth muscle dysfunction, including enlarged bladder, dilated gut, and hydronephrosis (Figure 4E). The bladders of ≈80% of *ACTA2*^R179H/+^ mice were markedly dilated, with reduced muscle wall thickness compared with WT mice. Hematoxylin & eosin staining of the WT small intestine revealed a well-preserved intestinal structure with densely organized villi. In contrast, the small intestines of ≈70% of *ACTA2*^R179H/+^ mice showed an increased lumen size, and the villi were disorganized, atrophied, or fragmented. In addition, the kidneys of ≈40% of *ACTA2*^R179H/+^ mice were larger with a thinner cortex compared with WT kidneys. The medullary regions of *ACTA2*^R179H/+^ kidneys were dilated and severely damaged, and hydronephrosis was apparent. We conclude that the *ACTA2*^R179H/+^ mice replicate the phenotypes observed in patients carrying the same sequence variant.

### Identification of a Smooth Muscle–Specific Promoter for AAV Delivery in Vivo

**Figure 5.**
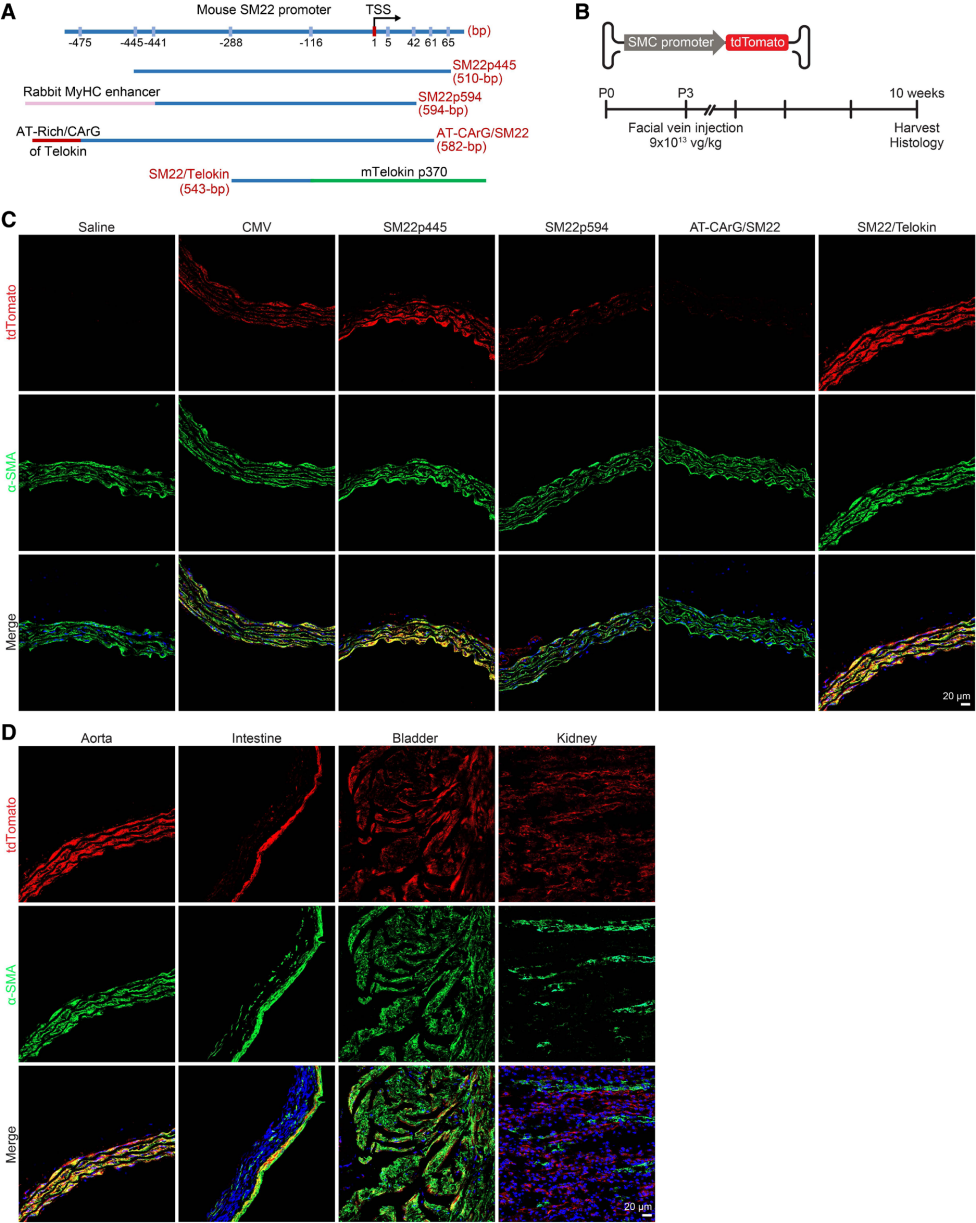
**Identification of an optimized smooth muscle–specific promoter. A**, Structures of SMC–specific promoters. **B**, Schematic of AAV construct and injection strategy to express the tdTomato reporter. **C**, Representative immunofluorescence staining of thoracic aorta shows tdTomato expression driven by the CMV promoter and a series of SMC-specific promoters. Mice injected with saline were used as a negative control. **D**, Representative immunofluorescence staining of thoracic aorta, small intestine, bladder, and kidney shows tdTomato expression driven by the SM22/Telokin promoter. Representative images reflect the most typical or consistent results observed across replicates. The aorta images for the SM22/Telokin promoter are the same in **C** and **D**. α-SMA indicates smooth muscle α-actin; AAV, adeno-associated virus; CMV, cytomegalovirus; SMC, smooth muscle cell; and TSS, transcription start site.

Although several promoters for vSMCs have been developed to study smooth muscle gene expression,^53–55^ few studies have attempted to transduce vSMCs in vivo using AAV.^56,57^ This may be attributable to the limited packaging capacity of AAV and the lack of suitable promoters for efficient vascular smooth muscle transduction. To ensure specific expression of the base editor within smooth muscle, we generated a series of AAV9 constructs expressing a tdTomato reporter driven by various smooth muscle–specific promoters (Figure 5A and 5B). We selected AAV9 because of its broad tissue tropism and its transduction in the aorta.^57^ We tested a series of promoters for their ability to direct strong and specific expression in smooth muscle, including a 510-bp mouse SM22 promoter fragment (−445 to +65; p445)^29^; a 594-bp chimeric promoter in which a rabbit MyHC enhancer was fused to the SM22 promoter fragment (−440 to +42; p594); a 582-bp chimeric promoter in which the telokin AT/CArG region was fused to the SM22 promoter fragment (−475 to +61; AT-CArG/SM22)^30^; and a 543-bp chimeric promoter in which a fragment of the SM22 promoter (−288 to −116) was fused to the telokin promoter fragment (−190 to +180; SM22/Telokin^30^) (Figure 5A). The cytomegalovirus (CMV) promoter (584-bp) was used as a positive control.

AAV virus at a dose of 9×10^13^ vg/kg was injected into WT mice at P3 through the facial vein (Figure 5B). Ten weeks after injection, aorta, intestine, bladder, kidney, heart, and liver tissues were harvested and stained for α-SMA and tdTomato to assess AAV transduction efficiency. Among the promoters tested, the chimeric mouse promoter SM22/Telokin showed the highest expression of the tdTomato reporter in both vascular and visceral smooth muscle (Figure 5C and 5D). Compared with the CMV promoter, the SM22/Telokin promoter exhibited minimal tdTomato expression in the liver or heart (Figure S8). We therefore selected the SM22/Telokin promoter for in vivo base editing applications.

### In Vivo Correction of Humanized *ACTA2*^R179H/+^ Mice Through Systemic AAV9 Delivery of Base Editing Components

**Figure 6.**
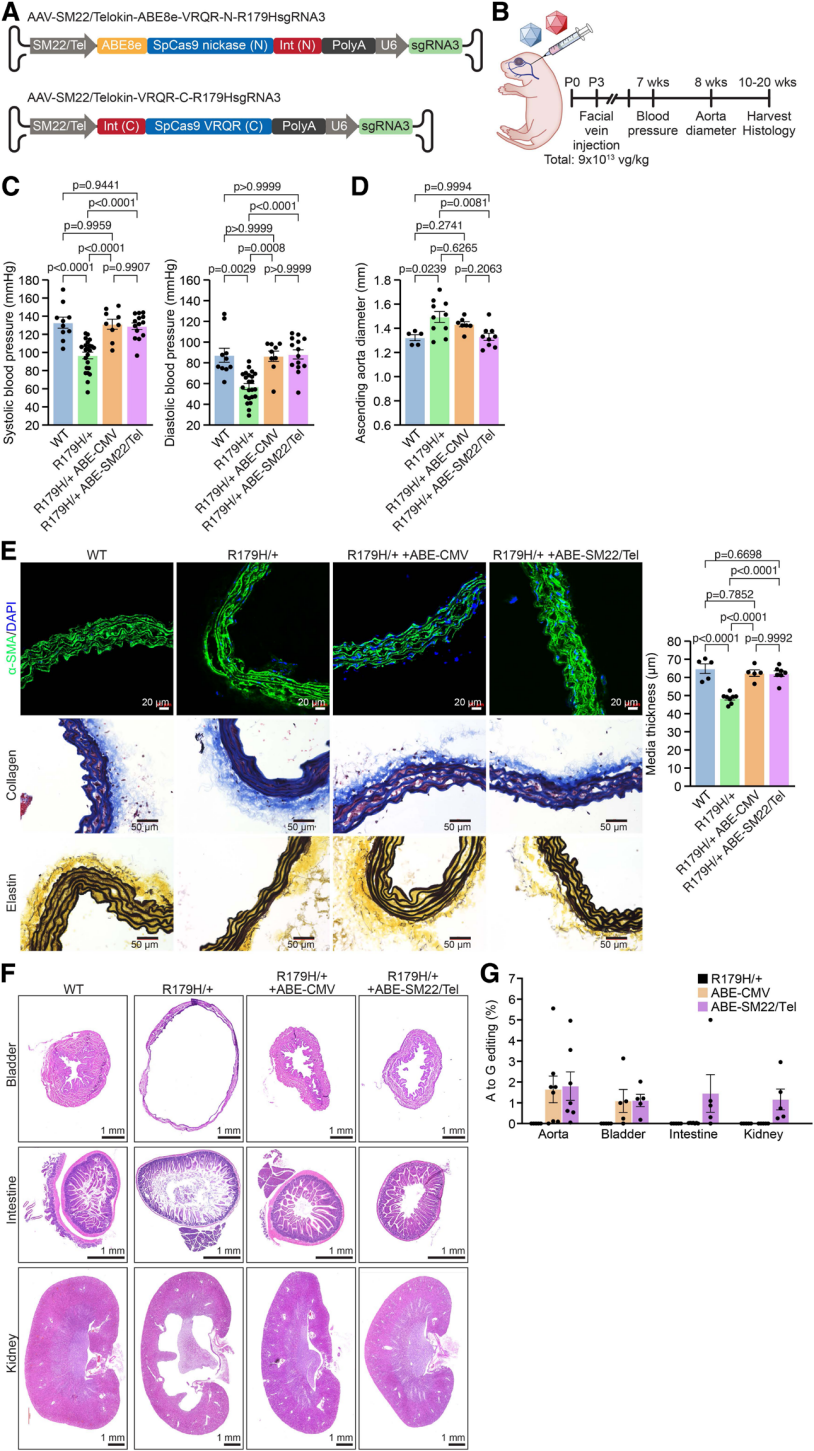
**Prevention of MSMDS by dual AAV9-mediated adenine base editing of *ACTA2*^R179H/+^ mice. A**, Schematic of the dual AAV9 ABE system encoding ABE8e-SpCas9-VRQR base editor halves under the control of the SM22/Telokin promoter and sgRNA3 under the control of the U6 promoter to target the human *ACTA2* p.R179H variant. **B**, Experimental outline for facial vein injection of *ACTA2*^R179H/+^ mice with saline or dual AAV9-mediated ABE at postnatal day 3 (P3) followed by blood pressure measurements, ultrasound measurements, and histological analysis. **C**, Systolic and diastolic blood pressure measurements of indicated mouse groups at 7 weeks of age (WT [n=10], R179H/+ [n=23], R179H/+ ABE-CMV [n=9], R179H/+ ABE-SM22/Tel [n=14]). **D**, Ascending aorta diameter measured by ultrasound of indicated mouse groups at 8 weeks of age (WT [n=5], R179H/+ [n=10], R179H/+ ABE-CMV [n=7], R179H/+ ABE-SM22/Tel [n=9]). **E**, Representative images of immunofluorescence staining, collagen staining, and elastin staining of the thoracic aorta and quantification of aortic media thickness of indicated mouse groups at 10 weeks of age (WT [n=5], R179H/+ [n=8], R179H/+ ABE-CMV [n=5], R179H/+ ABE-SM22/Tel [n=7]). **F**, Representative hematoxylin & eosin staining of bladder (top), small intestine (middle), and kidney (bottom) of indicated mouse groups at 20 weeks of age (WT [n=5], R179H/+ [n=4], R179H/+ ABE-CMV [n=3], R179H/+ ABE-SM22/Tel [n=4]). **G**, Percentage of adenine-to-guanine editing from aorta (R179H/+ [n=5], R179H/+ ABE-CMV [n=8], R179H/+ ABE-SM22/Tel [n=7]), bladder (R179H/+ [n=5], R179H/+ ABE-CMV [n=5], R179H/+ ABE-SM22/Tel [n=5]), small intestine (R179H/+ [n=5], R179H/+ ABE-CMV [n=5], R179H/+ ABE-SM22/Tel [n=5]), and kidney (R179H/+ [n=5], R179H/+ ABE-CMV [n=5], R179H/+ ABE-SM22/Tel [n=5]) of *ACTA2*^R179H/+^, AAV9-CMV-ABE–treated *ACTA2*^R179H/+^, and AAV9-SM22/Tel-ABE–treated *ACTA2*^R179H/+^ mice. The quantitative data are presented as mean±SEM. Statistical comparisons are based on 1-way ANOVA post hoc corrected by Tukey. Representative images reflect the most typical or consistent results observed across replicates. AAV indicates adeno-associated virus; ABE, adenine base editing; CMV, cytomegalovirus; MSMDS, multisystemic smooth muscle dysfunction syndrome; sgRNA, single guide RNA; SM22/Tel, SM22/Telokin; and WT, wild-type.

To evaluate whether ABE correction could rescue aortic defects and prevent the onset of MSMDS, we packaged expression cassettes encoding the ABE components, including ABE8e-SpCas9-VRQR and sgRNA3, into AAV vectors. Because the full-length base editor (≈5.1 kb) exceeds the packaging limit of a single AAV9 (≈4.7 kb), we split the base editor into 2 AAV9 vectors and used trans-splicing inteins to reassemble the base editor within cells during protein expression.^58^ Expression of the base editor was driven by the SM22/Telokin promoter to restrict its expression to SMCs and expression of sgRNA3 was under the control of the U6 promoter (Figure 6A). The CMV promoter was also included as a positive control (Figure S9).

We injected *ACTA2*^R179H/+^ pups at P3 through the facial vein with either saline or 4.5×10^13^ vg/kg of each AAV virus (total of 9×10^13^ vg/kg) (Figure 6B). BP measurements were conducted at 7 weeks of age. The results showed that saline-treated *ACTA2*^R179H/+^ mice had significantly lower systolic and diastolic BPs compared with WT mice (*ACTA2*^R179H/+^ 96.70±3.66 versus WT 132.89±6.13 mm Hg for systolic; *ACTA2*^R179H/+^ 57.10±2.89 versus WT 87.33±6.77 mm Hg for diastolic). In contrast, the systolic and diastolic BPs were normalized to WT levels in both AAV9-CMV-ABE–treated mice (131.14±5.54 and 86.40±4.99, respectively) and AAV9-SM22/Telokin-ABE–treated mice (129.00±3.66 and 88.11± 4.44, respectively) (Figure 6C).

Ultrasound measurements were conducted at 8 weeks of age to measure the aortic diameters. We observed a 13% increase in ascending aortic diameter in saline-treated *ACTA2*^R179H/+^ mice compared with WT controls (1.49±0.05 versus 1.32±0.03 mm). AAV9-CMV-ABE–treated *ACTA2*^R179H/+^ mice showed a slight reduction in aorta diameter compared with saline-injected *ACTA2*^R179H/+^ mice (1.43±0.02 versus 1.49±0.05 mm). AAV9-SM22/Telokin-ABE–treated *ACTA2*^R179H/+^ mice displayed aortic diameters similar to WT controls (1.33±0.03 mm versus 1.32±0.03 mm) (Figure 6D).

We next harvested aortas at 10 weeks of age and analyzed tissue sections by histological analysis (Figure 6E). Immunofluorescence staining for α-SMA demonstrated reduced media thickness in saline-injected *ACTA2*^R179H/+^ mice compared with WT mice (48.47±0.95 versus 64.82±2.65 µm). In contrast, both AAV9-CMV-ABE–treated (62.37±1.82 µm) and AAV9-SM22/Telokin-ABE–treated (62.07±1.56 µm) *ACTA2*^R179H/+^ mice showed similar media thickness to WT mice. In addition, increased collagen deposition in the media layer and elastin fiber fragmentation were observed in saline-injected *ACTA2*^R179H/+^ aortas, whereas AAV9-CMV-ABE–treated and AAV9-SM22/Telokin-ABE–treated aortas exhibited similar collagen and elastin staining to those of WT mice.

Angiotensin II (Ang II) contributes to the development of AAs, leading to increased wall thicknesses, dilation, and potential rupture of the aorta.^59^ To further evaluate the stability of ABE-corrected aortas under stress, we administered Ang II (1 μg·kg·min) to WT, saline-treated *ACTA2*^R179H/+^, and AAV9-SM22/Telokin-ABE–treated *ACTA2*^R179H/+^ mice for 2 weeks. Ultrasound measurements showed that saline-treated *ACTA2*^R179H/+^ mice had an increased ascending aortic diameter compared with WT mice (1.71±0.07 versus 1.54±0.02 mm), whereas AAV9-SM22/Telokin-ABE–treated *ACTA2*^R179H/+^ mice exhibited aortic diameters similar to WT mice (1.53±0.08 versus 1.54±0.02 mm) after Ang II infusion (Figure S10A). No obvious aneurysms were observed in WT and AAV9-SM22/Telokin-ABE–treated *ACTA2*^R179H/+^ mice after Ang II infusion; one saline-treated *ACTA2*^R179H/+^ mouse (1 out of 4) exhibited an obvious aneurysm throughout the thoracic and abdominal aorta, accompanied by thrombus formation, after 2 weeks of Ang II infusion (Figure S10B). Immunofluorescence and hematoxylin & eosin staining demonstrated increased thoracic and abdominal aortic wall thickness induced by Ang II in saline-treated *ACTA2*^R179H/+^ mice compared with WT mice (Figure S10C). In contrast, AAV9-SM22/Telokin-ABE–treated *ACTA2*^R179H/+^ mice showed thoracic and abdominal aortic wall thickness similar to WT mice after Ang II infusion. These results indicate that *ACTA2*^R179H/+^ mice are more vulnerable to Ang II infusion than WT mice. However, ABE correction mitigated this vulnerability, suggesting that ABE-corrected aortic tissues are capable of maintaining normal contractility and structure under stress conditions. Thus, correction of the pathogenic nucleotide in *ACTA2*^R179H/+^ mice by base editing was sufficient to rescue aortic structure and function.

The defects in visceral organs of *ACTA2*^R179H/+^ mice developed more slowly than those in the aorta, possibly because *ACTA2* is not the primary actin isoform in these tissues. We harvested intestine, bladder, and kidney at ≈20 weeks of age for histological analysis. As shown in Figure 6F, the saline-injected *ACTA2*^R179H/+^ mice exhibited a dilated small intestine with increased lumen size and disorganized villi. The bladder was highly dilated, and the kidneys displayed enlarged medullary regions. In contrast, these organs from AAV9-CMV-ABE–treated and AAV9-SM22/Telokin-ABE–treated *ACTA2*^R179H/+^ mice displayed morphologies similar to those of WT mice, indicating that ABE successfully mitigated these abnormalities.

To assess tissue-level gene editing efficiency, we collected aorta, intestine, bladder, and kidney samples from saline-treated, AAV9-CMV-ABE–treated, and AAV9-SM22/Telokin-ABE–treated *ACTA2*^R179H/+^ mice and evaluated on-target DNA editing efficiencies by deep amplicon sequencing. In AAV9-SM22/Telokin-ABE–treated *ACTA2*^R179H/+^ mice, the DNA editing efficiency at the target pathogenic adenine was 2.10±0.74% in the aorta (up to ≈5%), 1.11±0.29% in the bladder (up to ≈2%), 1.45±0.91% in the intestine (up to ≈5%), and 1.16±0.50% in the kidney (up to ≈3%). AAV9-CMV-ABE–treated *ACTA2*^R179H/+^ mice exhibited similar editing efficiencies in the aorta and bladder, with 1.65±0.65% in the aorta (up to ≈5.5%) and 1.09±0.55% in the bladder (up to ≈3%). However, these mice showed lower editing efficiencies in the intestine and kidney, with only 0.02±0.01% in the intestine and 0.001±0.001% in the kidney (Figure 6G). These modest efficiencies likely reflect the heterogeneity of cell types in these tissues, such that only a fraction of cells express the editors.

To evaluate the potential safety of the AAV treatment, we measured liver and kidney functions. Our findings revealed no significant elevation in aspartate aminotransferase, alanine aminotransferase, blood urea nitrogen, or creatinine levels in AAV9-ABE–treated *ACTA2*^R179H/+^ mice, suggesting minimal to no systemic toxicity (Figure S11).

These data suggest that our dual AAV9 ABE system safely and effectively corrects the pathogenic *ACTA2*^R179H^ nucleotide in genomic DNA, thereby preventing the onset of MSMDS.

## DISCUSSION

Precise base editing has shown efficacy in treating various diseases across different organs, including Duchenne muscular dystrophy,^15,23,60–63^ hypertrophic cardiomyopathy,^16,64^ dilated cardiomyopathy,^24^ sickle cell disease,^17,65^ and progeria.^57^ In this study, we explored the therapeutic potential of base editing for diseases originating in smooth muscle. We show that ABE can effectively correct an MSMDS-causing *ACTA2* sequence variant (c.536G>A) in human iPSC-SMCs, restoring contractile SMC phenotypes without inducing substantial bystander or off-target edits.

To translate these efficient in vitro gene editing outcomes to in vivo settings, we generated a humanized mouse model carrying the same *ACTA2* sequence variant and used AAV9-mediated delivery of the base editor. AAV9 is widely used for cardiac gene editing because of its high transduction in cardiac tissue and its clinical applicability. To achieve precise gene correction in SMCs, we screened and identified a chimeric mouse promoter, SM22/Telokin, which drives high tdTomato reporter expression in both vascular and visceral SMCs. As a positive control, we used the CMV promoter, a commonly used promoter in AAV vectors that drives high levels of gene expression across various tissues and has been shown to transduce vSMCs.^57^ Our results show that the chimeric SM22/Telokin promoter exhibited minimal expression in the liver and heart compared with the CMV promoter, highlighting the specificity of this combination of regulatory DNA sequences. Our findings demonstrated that both AAV9-SM22/Telokin-ABE and AAV9-CMV-ABE can alleviate aortic defects in vivo, highlighting a promising therapeutic approach for monogenic vascular diseases.

AAs and aortic dissections are severe vascular diseases that often lead to sudden death. Missense sequence variants in *ACTA2* account for 14% of inherited TAADs.^12^ We propose ABE-based gene editing in vSMCs as a novel and effective method for correction of *ACTA2* and other sequence variants in genes encoding vSMC contractile proteins. The F-actin disassembly caused by the *ACTA2* sequence variant results in loss of the SMC contractile phenotype. Weakness of the media layers of the aorta eventually leads to higher wall stress, which can induce aortic dilatation and aneurysm formation, eventually resulting in intramural hemorrhage, aortic dissection, or rupture. The optimal therapeutic window exists during the progression phase of the disease, before the onset of severe and irreversible damage, highlighting the importance of genetic screening at birth for families with known *ACTA2* sequence variants. Early detection could provide a critical window for gene-editing interventions, potentially preventing vSMC phenotypic switching and the formation of aneurysms.

Our study demonstrates that base editing can effectively correct vascular abnormalities in *ACTA2*^R179H/+^ mice. However, compared with our previous gene editing experiments in cardiac tissue, in which editing efficiency was ≈10 to ≈30%,^16,27^ the current efficiency in vSMCs was relatively low (up to 5% with the SM22/Telokin promoter and 5.5% with the CMV promoter in aorta). This outcome aligns with another report of a comparably low editing efficiency in vSMCs (5%) when AAV9 was injected at P3.^57^ Considering that the vSMC population only accounts for ≈10 to ≈20% of the whole aorta tissues, modest DNA editing may result in disproportionately large benefits at the RNA, protein, or tissue levels.^57^ Despite the relatively low editing efficiency, it is encouraging to observe substantial functional recovery in vivo after gene editing. We hypothesize that mosaic restoration of the contractile phenotype among vSMCs in the aortic arteries may be sufficient to delay the progression of aneurysm.^66^ Indeed, our results indicate that restoration of vascular structure and function can occur without complete correction of disease-causing genetic sequence variants. AAV9-SM22/Telokin-ABE treatment induced an editing efficiency up to 2% to ≈5% in the bladder, intestine, and kidney, but still resulted in effective rescue. One possible explanation is that the rescue observed in visceral organs is an indirect consequence of vascular correction.

We further evaluated the stability of ABE-corrected aortas under stress caused by Ang II. Previous studies on AA typically used *Apoe*^−/−^ mice on a high-fat diet with a 28-day infusion of Ang II (1 ug·kg·min), resulting in ≈80% AA incidence.^67^ In normocholesterolemic mice, the incidence of Ang II–induced AA is generally low (<20%) under the same conditions. In our study, we infused the mice on a normal diet with Ang II for 14 days. No obvious aneurysms were observed in WT or AAV9-SM22/Telokin-ABE–treated *ACTA2*^R179H/+^ mice after Ang II infusion. However, 1 saline-treated *ACTA2*^R179H/+^ mouse (1 out of 4) developed a clear aneurysm spanning the thoracic and abdominal aorta, accompanied by thrombus formation, after Ang II infusion. Aorta dilation and aorta wall thickness induced by Ang II were more obvious than in WT and AAV9-SM22/Telokin-ABE–treated *ACTA2*^R179H/+^ mice. These results indicate increased aortic sensitivity to Ang II in variant mice, whereas ABE correction preserves normal contractility and structure of the aorta under stress conditions. In this sense, the therapeutic value of base editing in treating human vascular diseases is further corroborated. Although *ACTA2* is not the predominant actin isoform in visceral organs, the *ACTA2* R179H sequence variant causes a severe, multisystemic disease, with complications affecting the gastrointestinal system, bladder, and kidney. We demonstrated that both AAV9-SM22/Telokin-ABE and AAV9-CMV-ABE treatments can ameliorate defects in the small intestine, bladder, and kidney.

In this proof-of-concept study, we demonstrate that base editing can successfully correct the *ACTA2* R179H sequence variant (c.536G>A) both in vitro and in vivo, highlighting its potential as a therapeutic approach for treating monogenic vascular diseases. Future studies will investigate whether this genetic correction is sufficient to prevent the onset of AAs and other MSMDS symptoms throughout life.

### Limitations

Whereas this study highlights the potential therapeutic application of adenine base editing to treat MSMDS and restore aortic smooth muscle function, several limitations should be noted. First, the *ACTA2*^R179H/+^ mouse model did not consistently replicate all abnormalities observed in human patients. The AAs and aortic dissections were milder than those seen in human patients. A possible explanation for this difference is that the hybrid genetic background of our mice may reduce the penetrance of the autosomal dominant effects associated with the *ACTA2* R179H sequence variant. It is possible that the *ACTA2*^R179H/+^ mice may develop more severe MSMDS symptoms over a longer timeframe, potentially mirroring the progression seen in human patients. It also seems likely that anatomic differences between mice and humans contribute to differences in aortic phenotypes. AAV, which was used to deliver the editing components, may cause adverse events. Although we did not observe systemic toxicity after AAV treatment, it will be of interest to test nonviral-based delivery methods in the future, such as the use of lipid nanoparticles or viral-like particles. In addition, the unexpectedly large benefits of modest DNA editing suggest that edited cells may influence tissue function disproportionately. Further studies are needed to elucidate the molecular basis of this phenomenon. Addressing these limitations in future studies will be imperative for translating these findings into safe and effective clinical applications.

## ARTICLE INFORMATION

### Acknowledgments

The authors thank Dr Rhonda Bassel-Duby and other members of the Olson Laboratory for discussions, Jose Cabrera for graphics, Dr Damir Alzhanov for assistance with iPSCs, the Boston Children’s Hospital Viral Core and the University of Michigan Vector Core for AAV9 production, the Children’s Medical Center Research Institute Sequencing Facility for performing the Illumina NextSeq sequencing, the UTSW McDermott Center Sanger Sequencing Core, and the Molecular Histopathology Core for help with histology.

### Sources of Funding

This study was supported by the National Institutes of Health (grants R01HL130253, R01HL157281, and P50HD087351), the Robert A. Welch Foundation (grant 1-0025), The Leducq Foundation Transatlantic Networks of Excellence, the British Heart Foundation Big Beat Challenge award to CureHeart (grant BBC/F/21/220106), and the American Heart Association (grant 25POST1372779) to Dr Ding.

### Disclosures

Dr Olson is a consultant for Vertex Pharmaceuticals, Tenaya Therapeutics, Prime Medicine, and Cardurion Pharmaceuticals. The other authors have no conflicts of interest.

### Supplemental Material

Checklist

Figures S1–S11

Tables S1–S2

## Supplementary Material
