# Supplementary material for: Genomic Editing of a Pathogenic Sequence Variant in ACTA2 Rescues Multisystemic Smooth Muscle Dysfunction Syndrome in Mice
Source: Circulation. 2025 May 16;152(7):465–83. doi: 10.1161/CIRCULATIONAHA.125.074218 (PMC12220935; doi:10.1161/CIRCULATIONAHA.125.074218)

SUPPLEMENTAL MATERIAL

Genomic editing of a pathogenic mutation in *ACTA2* rescues multisystemic smooth muscle dysfunction syndrome in mice

Qianqian Ding [1 2], PhD; Peiheng Gan [1 2], PhD, MBBS; Zhisheng Xu [1 2], PhD; Hui Li [1 2], PhD; Lei Guo [3], PhD; Camryn MacDonald [1 2], BSA; Wei Tan [1 2], MD; Efrain Sanchez-Ortiz [1 2], PhD; John R. McAnally [1 2], BS; Yu Zhang [1 2], PhD; Dileep Karri [1 2], MD, PhD; Lin Xu [3], PhD; Ning Liu [1 2], PhD & Eric N. Olson [1 2], PhD

Affiliations

1 Department of Molecular Biology, University of Texas Southwestern Medical Center, Dallas, TX, USA.

2 Hamon Center for Regenerative Science and Medicine, University of Texas Southwestern Medical Center, Dallas, TX, USA.

3 Quantitative Biomedical Research Center, Department of Population and Data Sciences, University of Texas Southwestern Medical Center, Dallas, TX, USA.

Correspondence to: Ning Liu, PhD, University of Texas Southwestern Medical Center, Molecular Biology, 5323 Harry Hines Blvd, Dallas, TX 75390-9148.

Email: ning.liu@utsouthwestern.edu

Eric Olson, PhD, University of Texas Southwestern Medical Center, Molecular Biology, 5323 Harry Hines Blvd, Dallas, TX 75390-9148.

Email: Eric.Olson@utsouthwestern.edu.

Supplemental Figures with Figure Legends

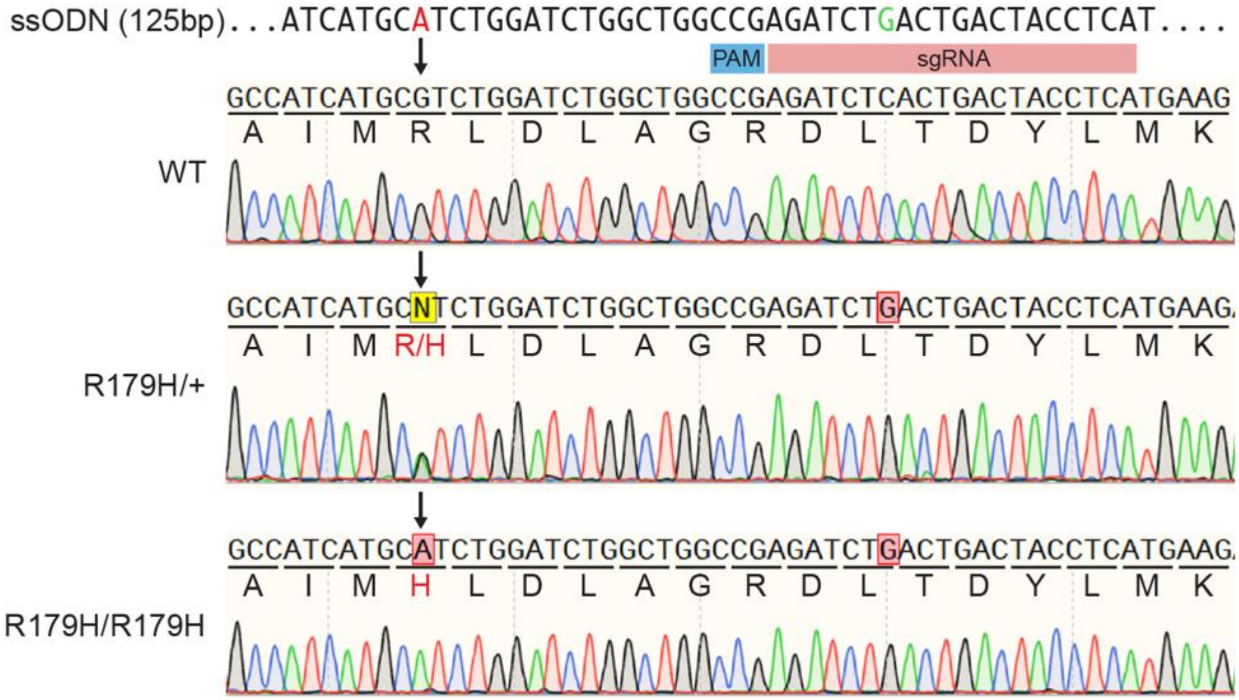

Figure S1. Generation of human iPSC lines carrying the R179H mutation. DNA and amino acid sequences of WT (top), heterozygous (R179H/+) (middle), and homozygous iPSC (R179H/R179H) lines (bottom). ssODN (125bp), sgRNA and PAM for homology-directed repair are indicated. A C-to-G silent mutation was introduced to avoid a second cut by the sgRNA. Arrows indicate the target nucleotide site.

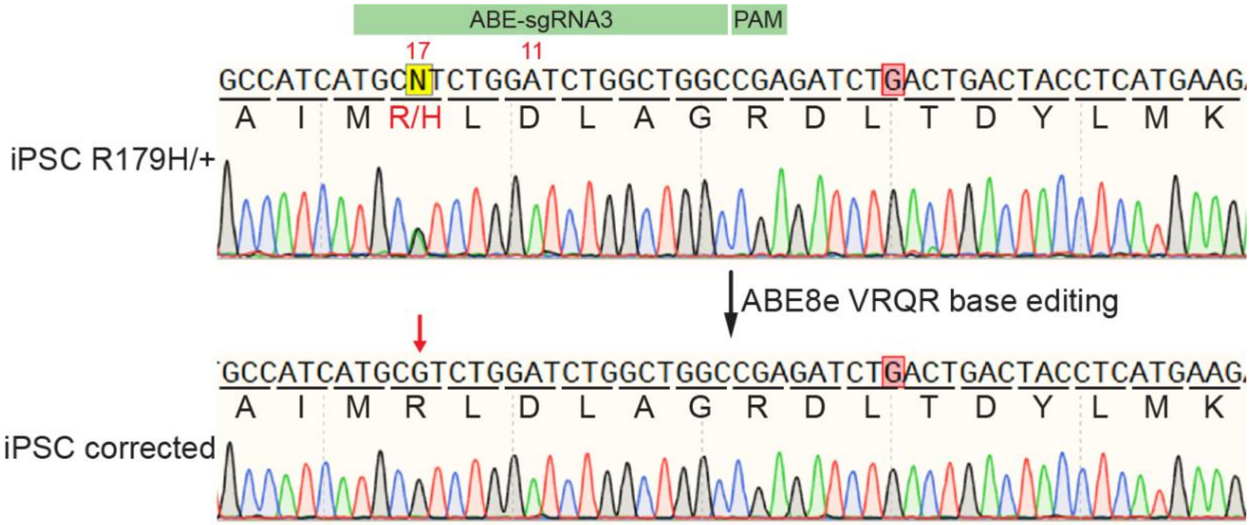

Figure S2. Generation of ABE-corrected iPSC line from heterozygous (R179H/+) iPSC line. The Sanger sequencing chromatogram displays the genomic region of *ACTA2* in both the heterozygous (R179H/+) and ABE-corrected iPSC lines derived from a single clone. Red arrow indicates the nucleotide after correction. sgRNAs and PAM are highlighted in green. The positions of the target A (A17) and the bystander A (A11) are indicated, with nucleotide counting beginning immediately 5′ of the PAM sequence.

A

Selected site	gRNA Sequence	PAM	Gene	Chromosome
On-target	TGCATCTGGATCTGGCTGGC (17, 11)	CGA	ACTA2	10
Off-target-1	TGAATAAGGATCAGGCTGGC	CGG	intron:SYT9	11
Off-target-2	TGCGCCTAGACCTGGCTGGC	CGG	intergenic:POTEG-CTD-2314B22.1	14
Off-target-3	GGAATCTGGGTCTGGCTGAC	GGG	COL4A6	X
Off-target-4	TGCATCCAGACCTGGCTTGC	CGG	intergenic:RALYL-LRRCC1	8
Off-target-5	AGAAGGTGGATCTGGCTGGC	TGG	intergenic:AC016768.1-AC012506.1	2
Off-target-6	TGAACCTGGATCTCCCTGGC	TGG	intergenic:LLNLR-304G9.1-PTPRS	19
Off-target-7	GGCACCTGGAGCTGGCAGGC	AGG	TCEB3C/TCEB3CL	18
Off-target-8	CCCAGCTGGGTCTGGCTGGC	TGG	intergenic:KIF26A-RP11-260M19.2	14
Adenine number	20 15 10 5 1			

B

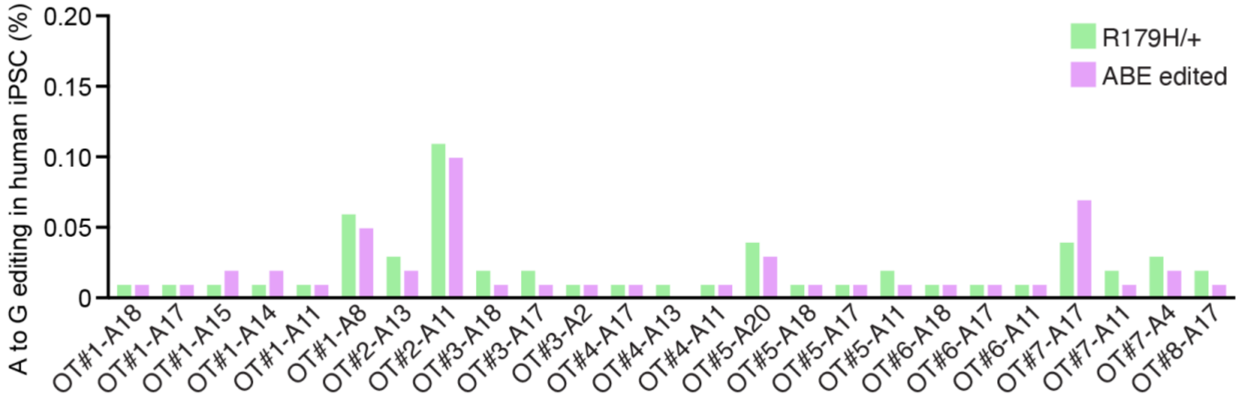

Figure S3. Computationally determined off-target sites for sgRNA3 with ABE8e-VRQR. (A) Genomic loci of eight candidate off-target (OT) sites. Nucleotides that differ are highlighted in yellow. Numbering of nucleotides in the protospacer starts with the nucleotide immediately 5′ of the PAM as nucleotide 1. **(B)** Deep amplicon sequencing to measure editing for all adenines within the protospacers of the top eight CRISPOR-identified candidate off-target loci in the edited cell pool.

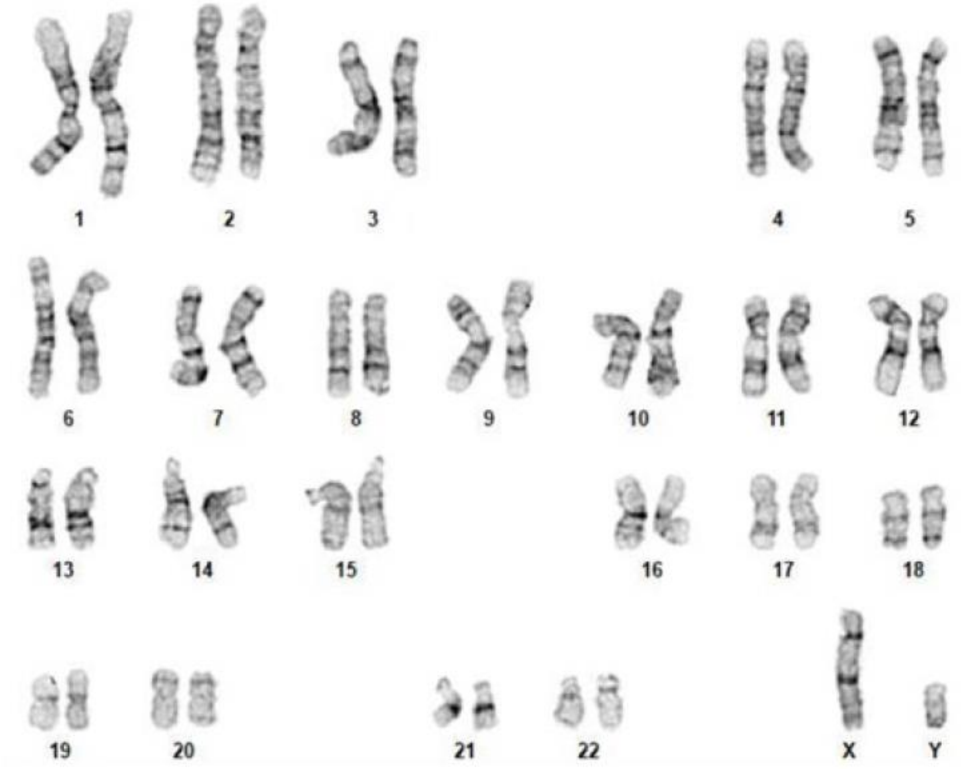

Figure S4. Normal karyotype was observed in *ACTA2*$^{R179H/+}$ iPSCs after ABE. Chromosome number is indicated beneath each chromosome pair.

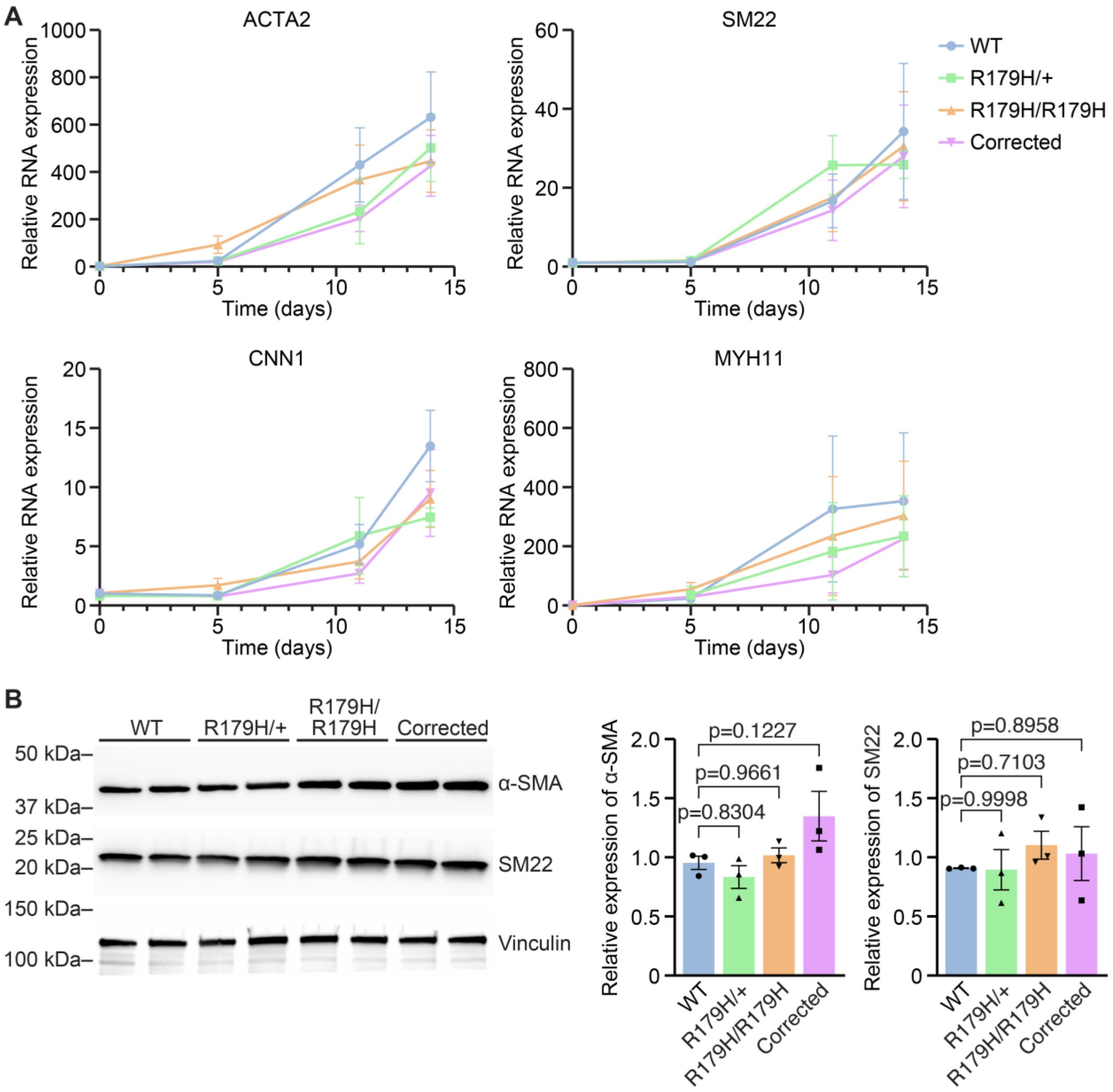

Figure S5. **(A)** Relative expression of the *ACTA2*, *SM22*, *CNN1*, and *MYH11* genes quantified by qRT-PCR in WT, R179H/+, R179H/R179H, and ABE-corrected iPSC-SMCs. **(B)** Representative western blots showing the expression of α-SMA, SM22, and Vinculin in WT, R179H/+, R179H/R179H, and ABE-corrected iPSC-SMCs. Expression of α-SMA and SM22 relative to Vinculin or GAPDH was quantified and normalized to WT. Quantitative data are presented as mean ± SEM.

Statistical comparisons are based on 1-way ANOVA post hoc corrected by Tukey and there is no significance among groups.

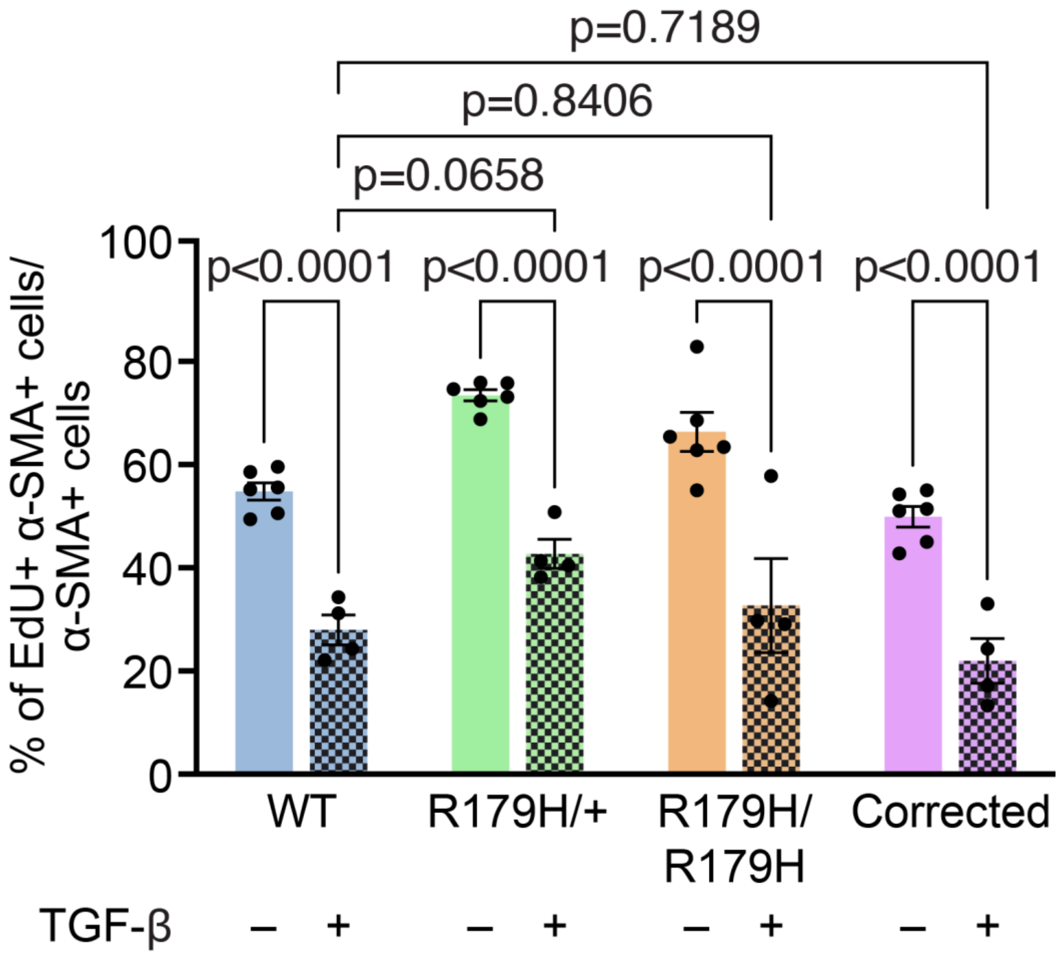

Figure S6. EdU assays of WT, R179H/+, R179H/R179H, and ABE-corrected iPSC-SMCs following TGF-β1 stimulation. The percentage of EdU^+ and $\alpha\text{-SMA}^+$ cells, relative to the total number of $\alpha\text{-SMA}^+$ cells, was quantified and displayed. The untreated samples are the same as shown in Figure 2A. Data are presented as mean ± SEM. Statistical comparisons are based on 2-way ANOVA post hoc corrected by Tukey.

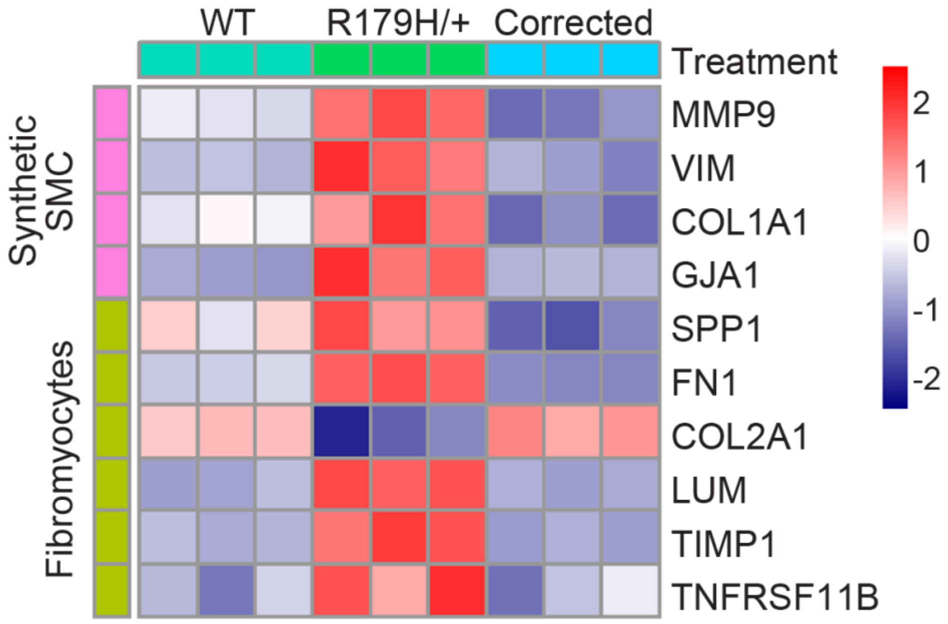

Figure S7. Heatmap of the differentially expressed gene markers for synthetic SMC and fibromyocyte cell populations in WT, R179H/+, and ABE-corrected R179H/+ iPSC-SMCs. Bulk RNA-seq is overlapped with scRNA-seq (PMID: 31359001) for the analysis.

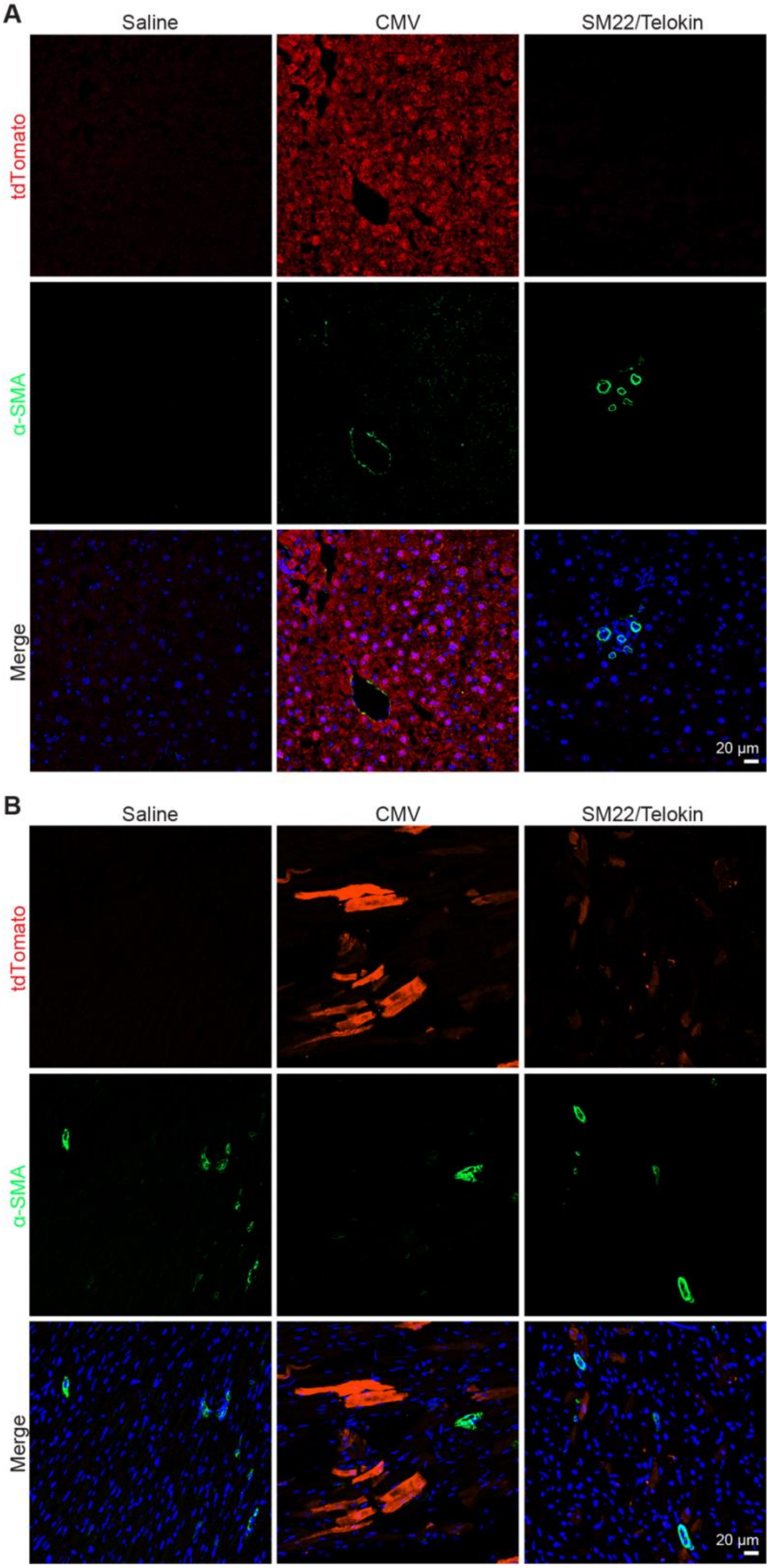
A
Saline
CMV
SM22/Telokin
tdTomato
α-SMA
Merge
20 μm
B
Saline
CMV
SM22/Telokin
tdTomato
α-SMA
Merge
20 μm

Figure S8. SM22/Telokin promoter drives minimal tdTomato expression in liver and heart. Representative IF staining of liver (A) and heart (B) show tdTomato expression driven by the CMV promoter and SM22/Telokin promoter. Mice injected with saline were used as a negative control.

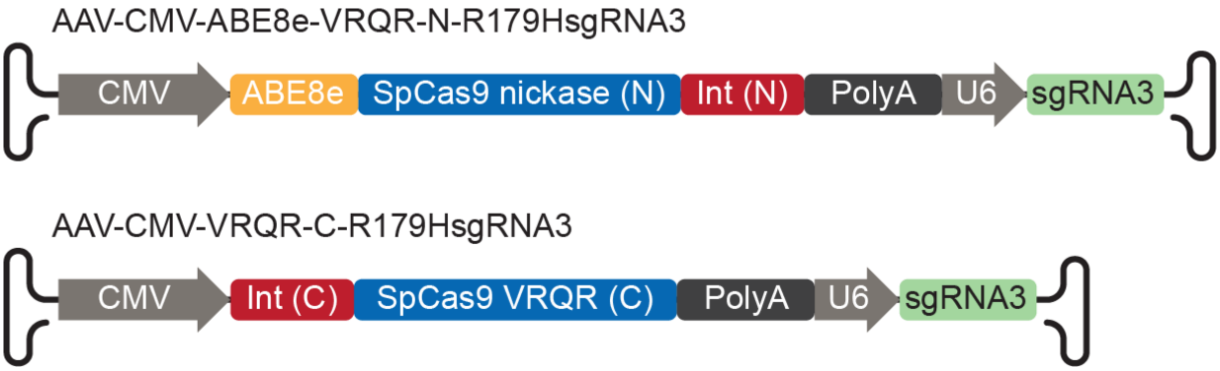

Figure S9. Schematic of dual AAV9 ABE system encoding ABE8e-SpCas9-VRQR base editor halves under control of the CMV promoter and sgRNA3 under control of the U6 promoter to target the human *ACTA2* p.R179H variant.

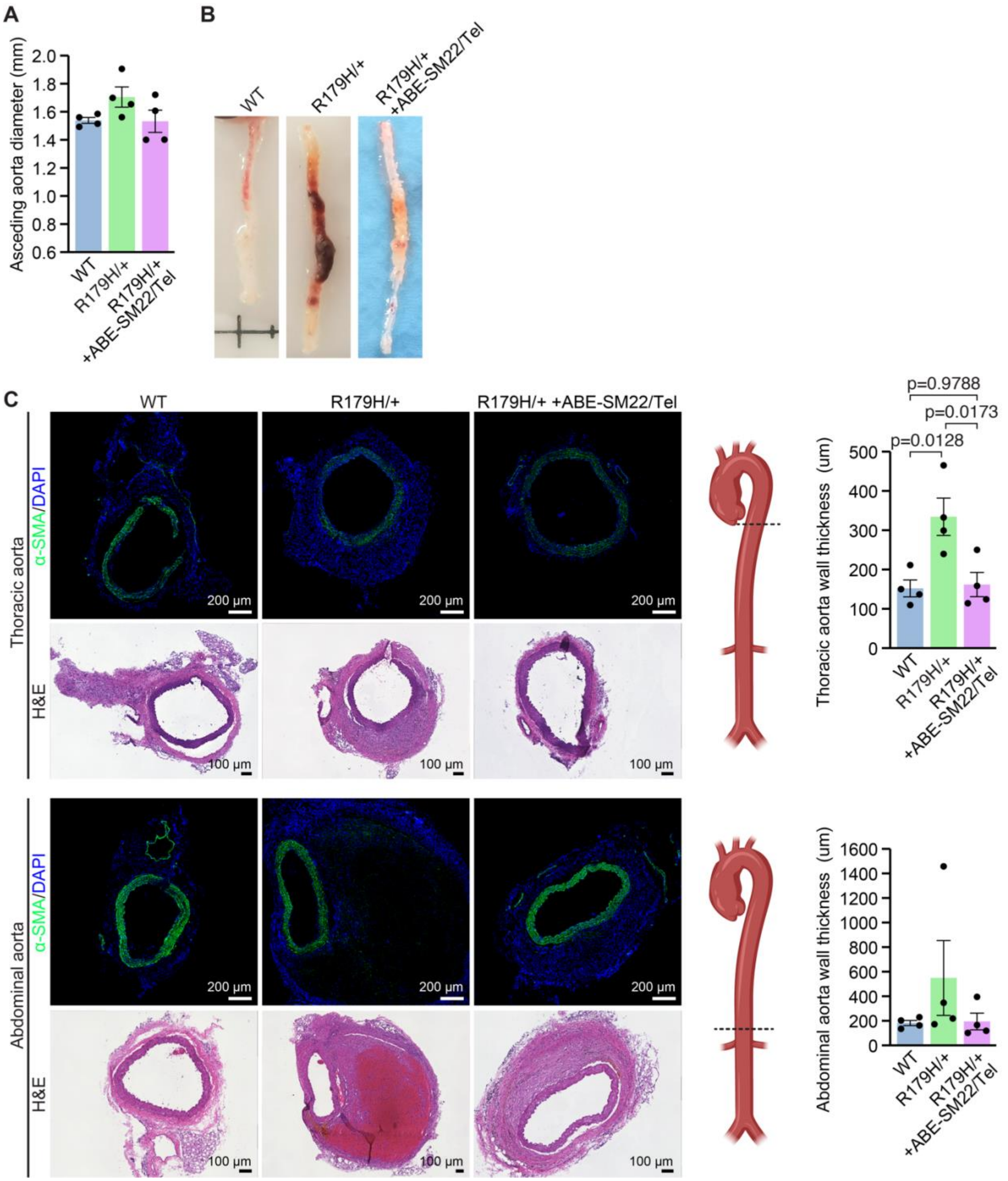

Figure S10. Aorta sensitivity to angiotensin II. (A) Ascending aorta diameters measured by ultrasound of the indicated mouse groups after AngII infusion. (B) WT, *ACTA2*$^{R179H/+}$, and AAV9-SM22/Tel-ABE-treated *ACTA2*$^{R179H/+}$ mice were infused with Ang II for 2 weeks and

representative aortas are shown. (C) IF and H&E staining were performed on thoracic and abdominal aortas from WT, *ACTA2*$^{R179H/+}$, and AAV9-SM22/Tel-ABE-treated *ACTA2*$^{R179H/+}$ mice infused with Ang II. The schematic diagram of the aorta indicates the location where sections were taken for the histological analysis of the aorta. Quantification of aortic wall thickness of indicated mouse groups after AngII infusion is also shown. n=4. The quantitative data are presented as mean ± SEM. Statistical comparisons are based on 1-way ANOVA post hoc corrected by Tukey.

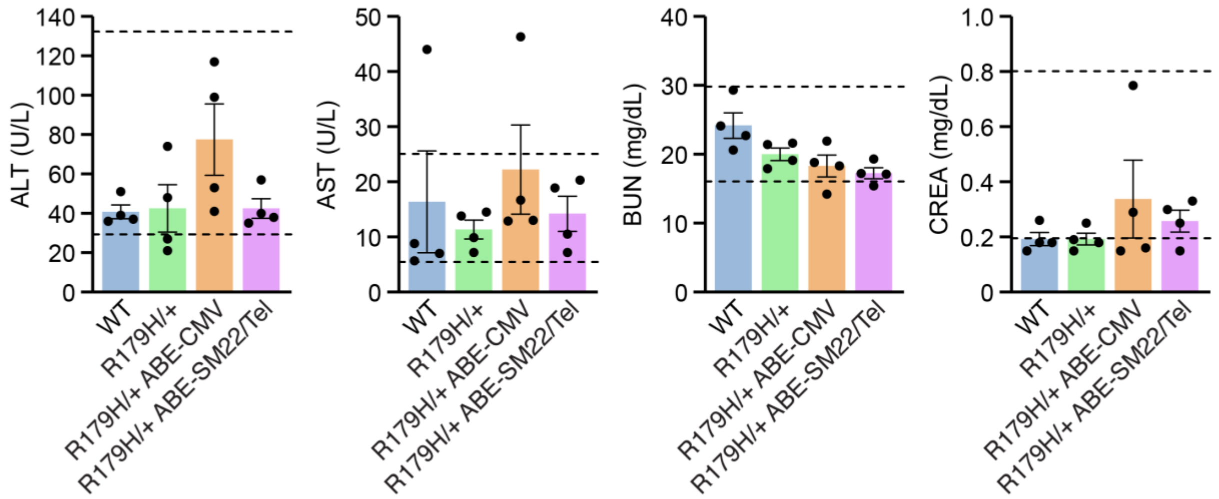

Figure S11. Liver and kidney toxicity. The effects of ABE treatment on ALT, AST, BUN and CREA levels in serum were evaluated in 6-month-old mice. The dashed lines represent the normal range for each measurement. Data were expressed as the mean ± SD (n = 4).

Supplemental Tables

Table S1. Base editing single guide RNAs (sgRNAs) for correction of the *ACTA2* R179H mutation.

Base editing sgRNA	sgRNA sequence	PAM
sgRNA1	ATCATGCATCTGGATCTGGC	TGG
sgRNA2	TCATGCATCTGGATCTGGCT	GGC
sgRNA3	TGCATCTGGATCTGGCTGGC	CGA

Table S2. List of primers and single-stranded oligodeoxynucleotides used in this study.

Primer function	Primer name	Primer sequence
hiPSC ACTA2 DNA amplification	hACTA2-F1	CCTCTCCTATCACATGCCCTGT
	hACTA2-R1	ACTGACTAATGCATAACACTGGGT
hiPSC ACTA2 DNA sequence	hACTA2-F1	AGGCTCGGAAATTCCCTCT
	hACTA2-R1	TGAGGATGGTCCTGGAAGTT
Mouse Acta2 DNA amplification	mActa2-F1	CAAGCCCTAGCTCTGACCTATCC
	mActa2-R1	CGCTGTGAGCTCAGGAACTG
Mouse Acta2 DNA sequence	mActa2-F2	GCACATTCTTTCTTCCCTTGA
	mActa2-R2	GGACAGCCCTCCTTCTGATA
Off-target	OT1-F	AGGATGCCTCATGGGTCAAT
	OT1-R	CCTGCATTTGCCTGGACCTA
	OT2-F	CGTGCTGTCCTTGTACACCT
	OT2-R	AGTCCAGGGCAACATAGCAC
	OT3-F	ATGGTGATGTCCTGGCTGTG
	OT3-R	TCTCCTTCCAGGCTTTGCTG
	OT4-F	TGGATTCCAGTGGCAGGTTC
	OT4-R	TCCAGCACAGCTTCTCCTTG
	OT5-F	TGCTCAGTGTCCTCAGGGTA
	OT5-R	GTCCACTTGAGCCCATCACT
	OT6-F	CTCAGAGGCCTTGCAGAGAC
	OT6-R	TCCCAGAGGATGCTTTGCTG
	OT7-F	CCCTTCCAGAACGGGTTCAA
	OT7-R	AAACCCAGAAGCACTCTCCG
	OT8-F	AGGTCATCACAGCACATGGG
	OT8-R	CTAAACGTCTGGCCACAGGT
Primers for qPCR	hACTA2-qPCR-F	ACGATGCTCCCAGGGCTGTT
	hACTA2-qPCR-R	TGCTCTGTGCTTCGTCACCCA

	hMYH11-qPCR-F	AGACACAAGTATCACGGGAGAGC
	hMYH11-qPCR-R	TGCGGATGAATTTGCCGAATCGT
	hCalponin 1 (CNN1)-qPCR-F	GCTGGAGAACATCGGCAACTTCAT
	hCalponin 1 (CNN1)-qPCR-R	TTCCTTTCGTCTTCGCCATGCT
	hTransgelin (SM22)-qPCR-F	CATCAAGACTGACATGTTCC
	hTransgelin (SM22)-qPCR-R	CTCTGTGAATTCCCTCTTATG
	hGAPDH-qPCR-F	GTCTCCTCTGACTTCAACAGCG
	hGAPDH-qPCR-R	ACCACCCTGTTGCTGTAGCCAA
	mIL-1b-qPCR-F	GCAACTGTTCCTGAACTCAACT
	mIL-1b-qPCR-R	ATCTTTTGGGGTCCGTCAACT
	mTNF-α-qPCR-F	CCCTCACACTCAGATCATCTTCT
	mTNF-α-qPCR-R	GCTACGACGTGGGCTACAG
	mIL6-qPCR-F	ACCACTTCACAAGTCGGAGG
	mIL6-qPCR-R	TCAGAATTGCCATTGCACAAC
	mIFNG-qPCR-F	CAGCAACAGCAAGGCGAAAAAGG
	mIFNG-qPCR-R	TTTCCGCTTCCTGAGGCTGGAT
Generating iPSCs		
Generating mouse model	mActa2-R179H-F	GATCTAATACGACTCACTATAGGTGAGGTAGTCGGTGAGATCTGTTTTAGAGCTAGAAAT
	mActa2-R179H-R	AAAAAAGCACCGACTCGGTGC
	ssODN	tacagcatacttaattgttccccagGTATTGTGCTGGACTCTGGAGATGGTGTGACTCACAACGTGCCTATCTATGAGGGCTATGCCCTGCCTCATGCCATCATGCaTCTGGAtcTGGCTGGCCGAGATCTCACCGACTACCTCATGAAGATCCTGACTGAGCGTGGCTATTCCTTCGTGACTACTGgtgaggctcgg
In vivo editing efficiency	mActa2-Deep amplicon-F	TCG TCG GCA GCG TC AGA TGT GTA TAA GAG ACA G gcacattctttcttcccttga

	mActa2-Deep amplicon-R	GTC TCG TGG GCT CGG AGA TGT GTA TAA GAG ACA Gggacagccctccttctgata
Barcode primers for Deep amplicon sequence	Universal barcode-F	AATGATACGGCGACCACCGAGATCT ACACTCGTCGGCAGCGTC
	Universal barcode-R	CAAGCAGAAGACGGCATACGAGATN NNNNNGTCTCGTGGGCTCGG

Full unedited gel for Figure 2D. Red box indicates bands used for quantification and representative images

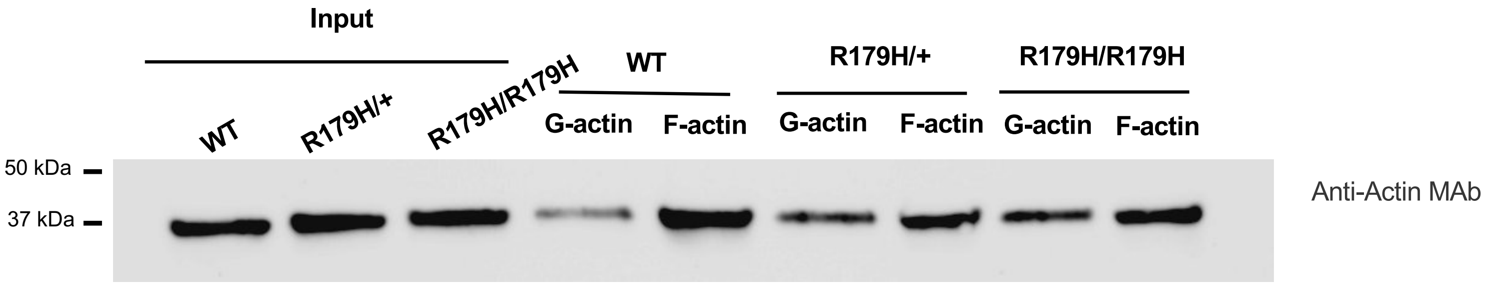

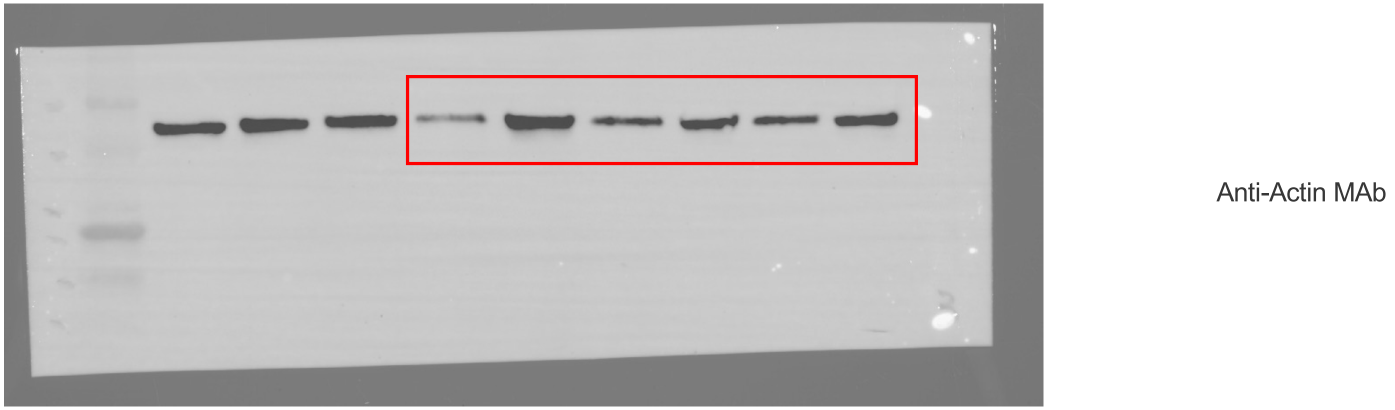

Full unedited gel for Figure 2D. Yellow box indicates bands used for quantification. Red box indicate bands used for quantification and representative images

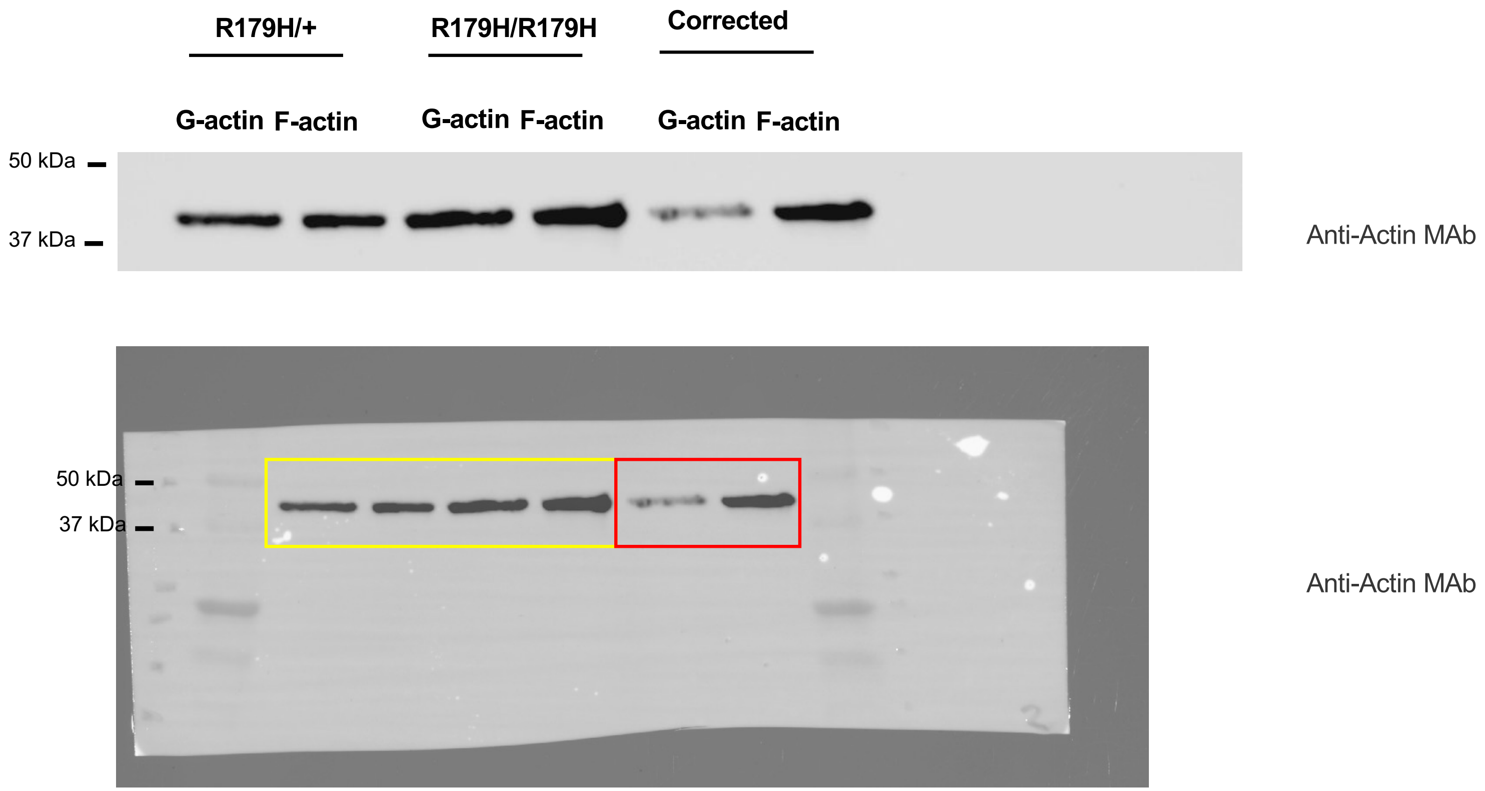

Full unedited gel for Figure 2D. Yellow box indicates bands used for quantification

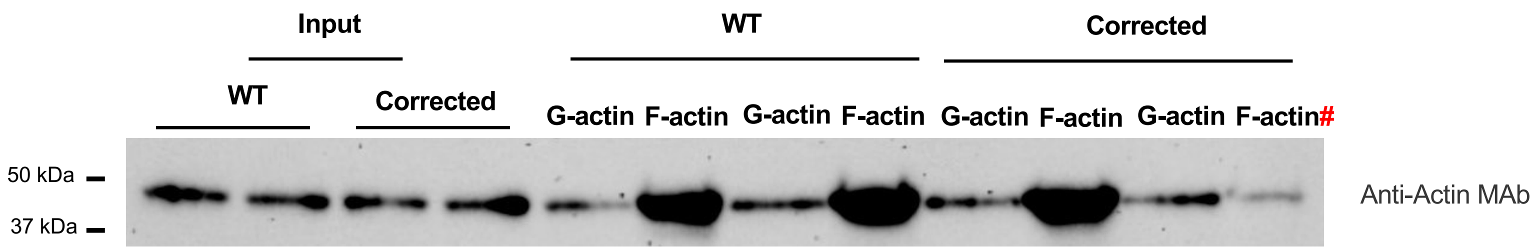

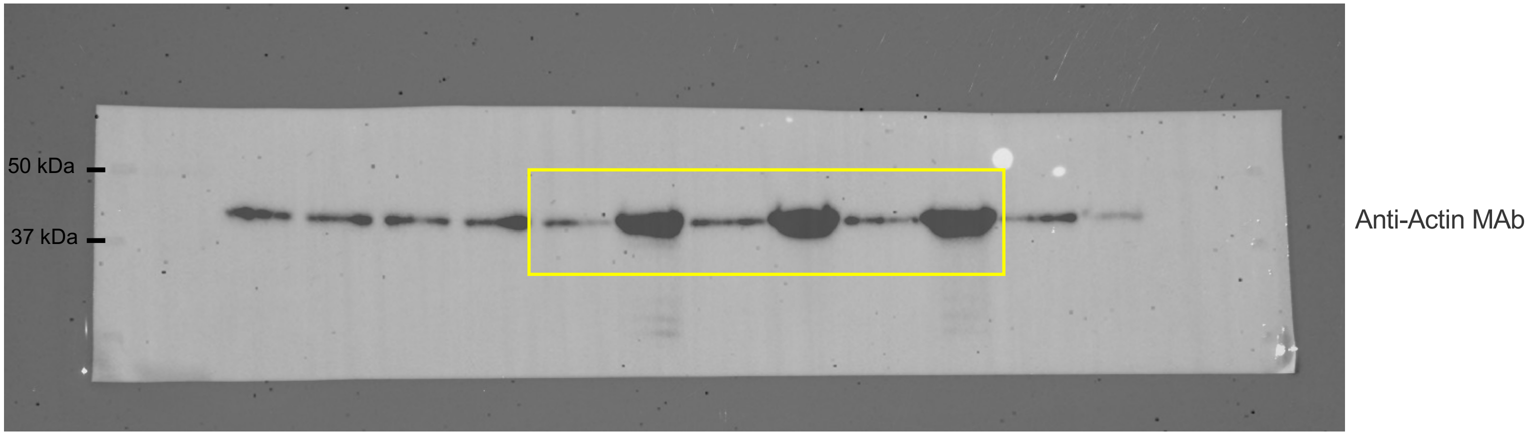

#: Some of the sample was lost during the loading process and have been excluded from quantification

Full unedited gel for Figure 2D. Yellow box indicate bands used for quantification

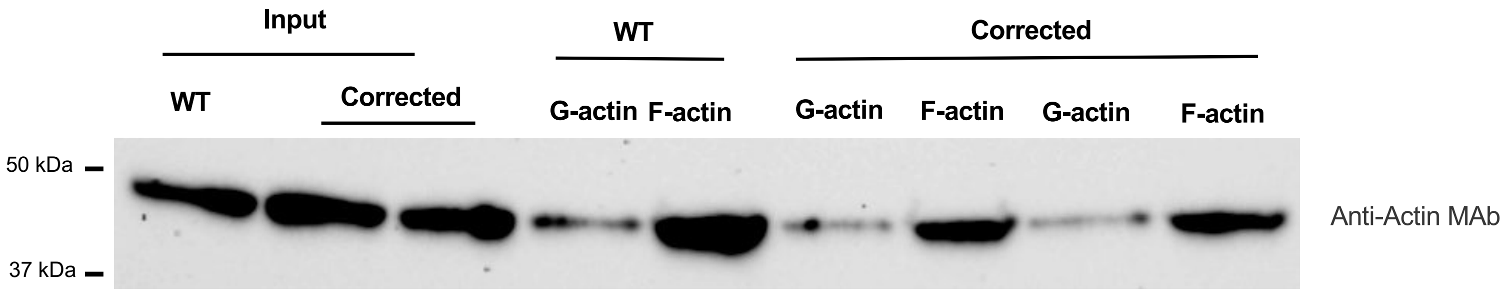

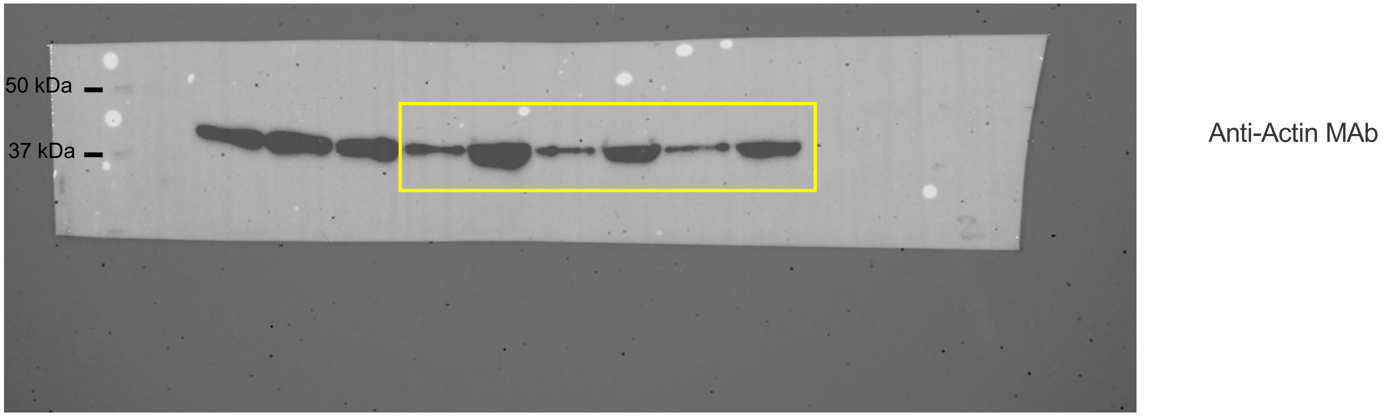

Full unedited gel for Figure 2D. Yellow box indicates bands used for quantification

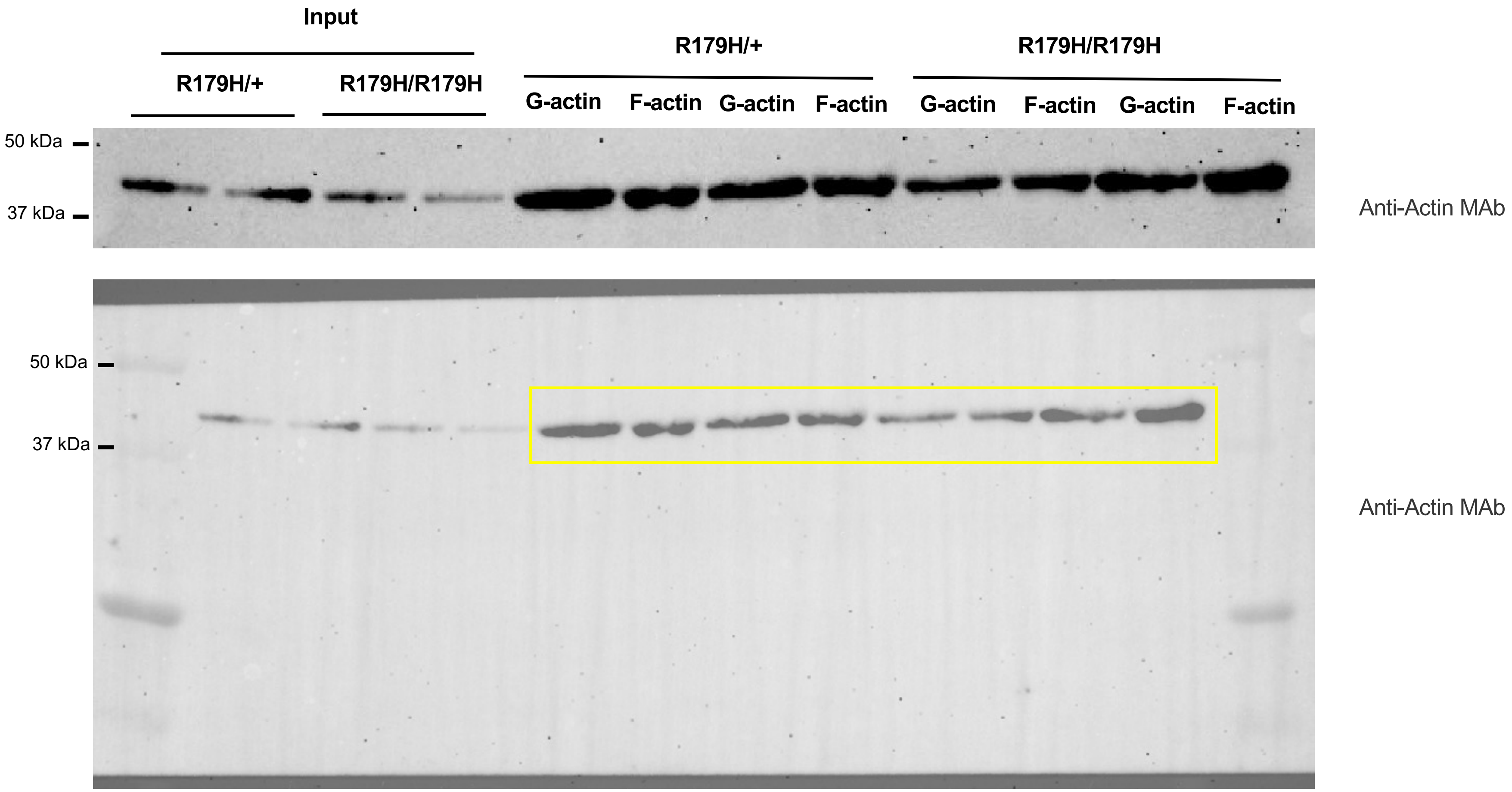

Full unedited gel for Figure S5B. Red boxes indicate bands used for quantification and representative images

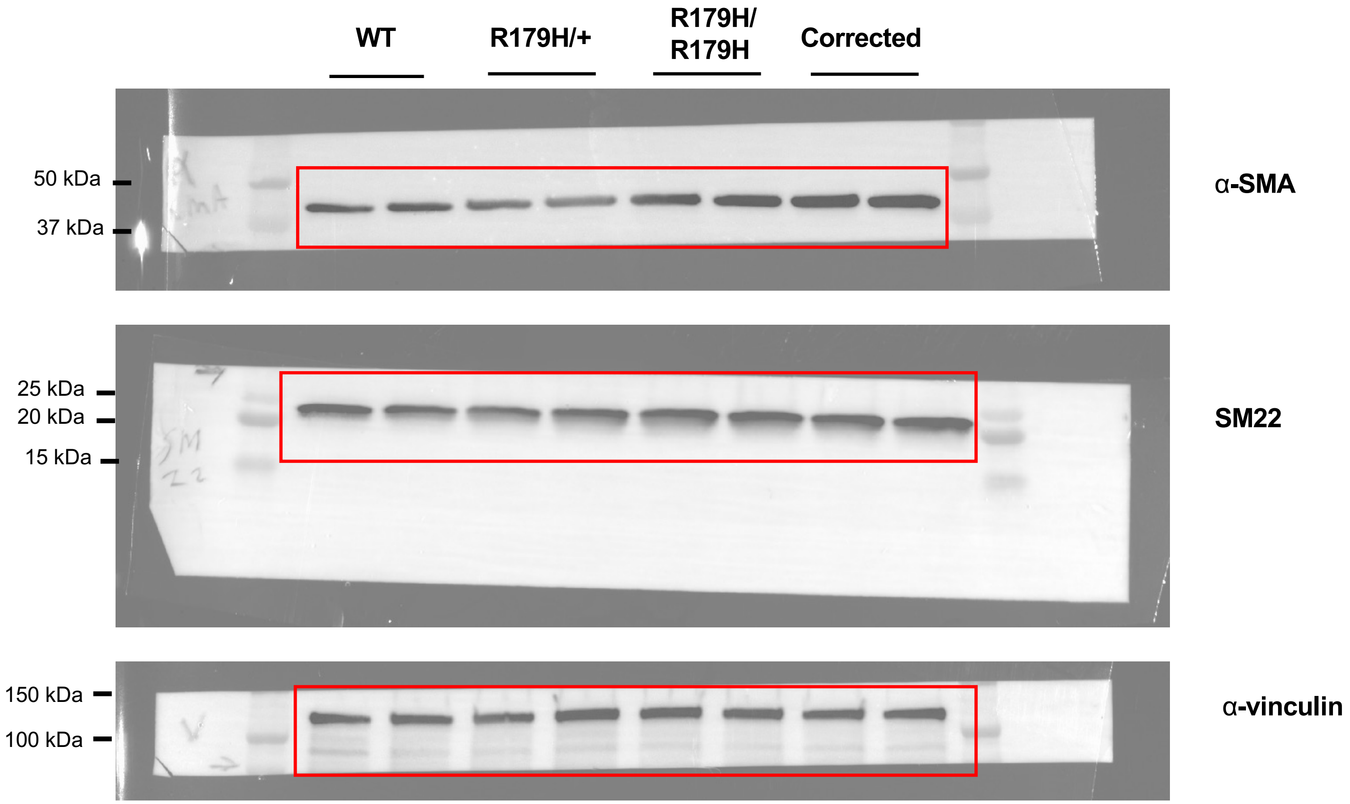

Full unedited gel for Figure S5B. Yellow boxes indicate bands used for quantification

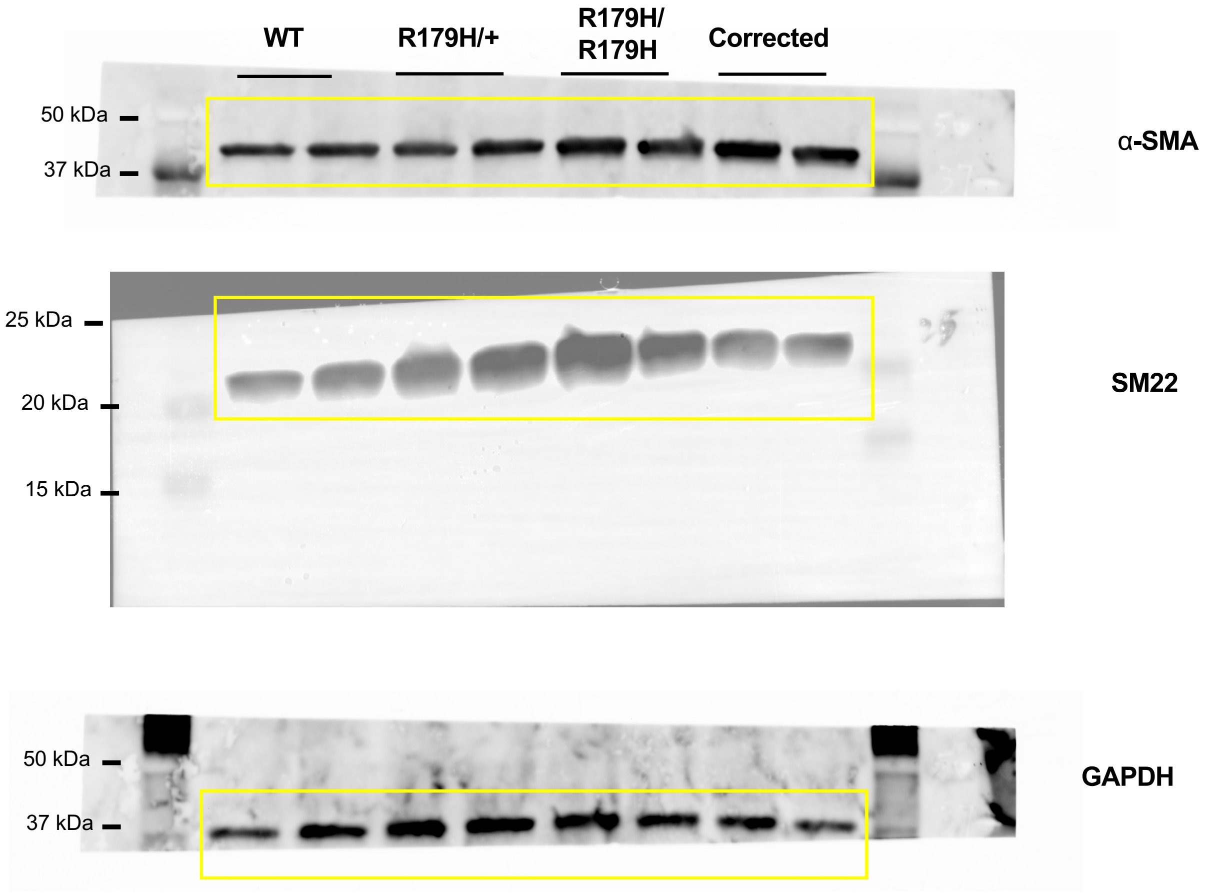

Supplement: Supplementary file 1 [file cir-152-465-s001.pdf]
